# Bending the curve: Simple but massive conservation action leads to landscape-scale recovery of amphibians

**DOI:** 10.1073/pnas.2123070119

**Published:** 2022-10-10

**Authors:** Helen Moor, Ariel Bergamini, Christoph Vorburger, Rolf Holderegger, Christoph Bühler, Simon Egger, Benedikt R. Schmidt

**Affiliations:** ^a^Swiss Federal Institute for Forest, Snow and Landscape Research WSL, Zürcherstrasse 111, CH-8093 Birmensdorf, Switzerland;; ^b^Eawag, Überlandstrasse 133, CH-8600 Dübendorf, Switzerland;; ^c^Department of Environmental Systems Science, ETH Zurich, CH-8092 Zurich, Switzerland;; ^d^Hintermann and Weber, Austrasse 2a, CH-4253 Reinach, Switzerland;; ^e^Sektion Natur and Landschaft, Kanton Aargau, Entfelderstrasse 22, CH-5001 Aarau, Switzerland;; ^f^info fauna karch, Bellevaux 51, CH-2000 Neuchâtel, Switzerland;; ^g^Department of Evolutionary Biology and Environmental Studies, University of Zurich, Winterthurerstrasse 190, CH-8057 Zürich, Switzerland

**Keywords:** amphibian decline, evidence-based conservation, freshwater biodiversity, recovery, conservation management

## Abstract

Success stories are rare in conservation science, hindered also by the research-implementation gap, where scientific insights rarely inform practice and practical implementation is rarely evaluated scientifically. Amphibian population declines, driven by multiple stressors, are emblematic of the freshwater biodiversity crisis. Habitat creation is a straightforward conservation action that has been shown to locally benefit amphibians, as well as other taxa, but does it benefit entire amphibian communities at large spatial scales? Here, we evaluate a landscape-scale pond-construction program by fitting dynamic occupancy models to 20 y of monitoring data for 12 pond-breeding amphibian species in the Swiss state Aargau, a densely populated area of the Swiss lowlands with intensive land use. After decades of population declines, the number of occupied ponds increased statewide for 10 out of 12 species, while one species remained stable and one species further declined between 1999 and 2019. Despite regional differences, in 77% of all 43 regional metapopulations, the colonization and subsequent occupation of new ponds stabilized (14%) or increased (63%) metapopulation size. Likely mechanisms include increased habitat availability, restoration of habitat dynamics, and increased connectivity between ponds. Colonization probabilities reflected species-specific preferences for characteristics of ponds and their surroundings, which provides evidence-based information for future pond construction targeting specific species. The relatively simple but landscape-scale and persistent conservation action of constructing hundreds of new ponds halted declines and stabilized or increased the state-wide population size of all but one species, despite ongoing pressures from other stressors in a human-dominated landscape.

## Significance

The global decline of amphibians is part of the global freshwater biodiversity crisis. In human-dominated landscapes, amphibian population declines are driven by multiple stressors. A better understanding of the benefits of conservation action can contribute to the halting and reversal of population declines. Our analysis of 20 y of monitoring data shows that the large-scale construction of hundreds of new ponds in northern Switzerland has halted or even reversed declining trends for the majority of amphibian species, including multiple Red-Listed species undergoing declines at the national level. This conservation success suggests that increasing habitat availability benefits threatened amphibian species despite the continued presence of stressors known to negatively affect populations.

In the Anthropocene, reports on the loss of biodiversity abound (1–3). The dual task of conservation science is to describe and explain declines and to provide the tools to halt and reverse the erosion of biodiversity from local to global scales (4, 5). Large-scale trend assessments frequently focus on the description and explanation of biodiversity decline but often do not establish direct links to conservation action (6–9). However, testing practical solutions to conservation problems remains important (10, 11). As Godet and Devictor showed, about a third of conservation-science articles discuss solutions, but the majority of these are only proposals and few actually test whether a solution worked (11). While some studies have shown that conservation action has positive effects on regional wildlife (4), many are proof-of-concept studies of local populations (12, 13), and we do not know whether their large-scale implementation is possible and would lead to ultimate conservation success (large-scale recovery of species; (14–16)). This is unfortunate because it is imperative to “bend the curve” toward the recovery of biodiversity (17).

The decline of wetlands and freshwater biodiversity continues to outpace declines seen in marine or terrestrial systems and has been dubbed “the ultimate conservation challenge” (18, 19). The decline of amphibians is a prime example of the freshwater biodiversity crisis because of its magnitude and the variety of causes (6, 20, 21). Many stressors have been described that negatively impact amphibians (e.g., habitat loss and degradation, emerging pathogens, pollution, climate change, roads, exploitation for human consumption, and invasive species; (20)), but there is no single most important stressor. Rather, every population is exposed to an idiosyncratic set of local stressors (9). Many stressors, such as climate change, emerging pathogens, or invasive species, are hard to mitigate (20). In contrast, habitat loss and degradation are important stressors that can, at least in principle, be alleviated relatively simply through habitat creation and management (22–24). Pond creation supports many aquatic and terrestrial taxa as well as ecosystem services (25). While there is ample evidence that restored and newly created ponds are readily colonized by amphibians (22, 23), it has not yet been shown that pond construction leads to landscape-scale recovery of amphibian communities (26).

In Switzerland, both rare and common amphibians declined strongly in the past and either continue to decline to this day or their populations linger at low levels (27–29). In the Swiss state Aargau, concern about these declines motivated a large-scale, ongoing pond-construction program (30). The state Aargau is a densely populated, highly urbanized landscape with managed forests and intensive farming, which is highly fragmented by traffic infrastructure and where few patches of natural ecosystems remain (31) (*SI Appendix*, Fig. S1). About 20% of the agricultural area is actively drained by drainage pipes (32). Pond-breeding amphibians are further affected by multiple stressors, such as nonnative fish, the amphibian chytrid fungus *Batrachochytrium dendrobatidis*, and pesticides (33–35). In this landscape, hundreds of ponds were added to the existing set of ponds and a monitoring program accompanied these measures.

Here, our aim was to evaluate whether this massive conservation effort benefitted declining amphibian species and whether negative trends could be reversed. To this end, we fitted dynamic occupancy models accounting for imperfect detection and observer bias to 20 y of monitoring data for all 12 pond-breeding amphibian species in the state Aargau (36). The data encompass thousands of surveys in 856 ponds, 422 of which were newly constructed between 1991 and 2019, distributed across five regions (Rheintal, Aaretal, Reusstal, Suhretal, and Wiggertal; *SI Appendix*, Fig. S2). The regions are geographically distinct and follow major river valleys separated by hills. We assumed that each region accommodates a distinct metapopulation, potentially connected by dispersal within (mean interpond distance within regions in 1999 was 420–800 m) but relatively isolated between regions. The 12 studied species included all extant pond-breeding amphibians (the stream-breeding salamander *Salamandra salamandra* was not part of the conservation and monitoring program). They comprised eight anurans (toads and frogs) and four newts with different life histories, habitat requirements, and dispersal capacities (*SI Appendix* S1). Eight species are on the national Red List (27), and one is an invasive species (*SI Appendix*, Table S1). Our analyses aimed (i) to describe the trajectories of Nos. of occupied ponds across and within five major regions in the Swiss state Aargau, (ii) to assess whether and how the colonization of new ponds contributed to changes in metapopulation sizes, (iii) to test whether colonization-persistence dynamics differed between existing (i.e., old) and newly constructed ponds, and (iv) to identify characteristics of the ponds that were important for colonization.

## Results and Discussion

### State-Wide and Regional Trends in the No. of Occupied Ponds.

To assess changes in state-wide population sizes, we compared the estimated number of ponds across the five regions occupied in 2019 and 1999 and classified differences into increases, stable Nos., or declines (based on the proportion of the posterior distribution of differences below or above zero with a threshold of 90% certainty). At the scale of the entire state, the number of occupied ponds increased for 10 of the 12 species from 1999 to 2019, did not change for *Alytes obstetricans*, and declined for *Epidalea calamita* (*SI Appendix*, Table S2 and Fig. S3).

**Fig. 1.**
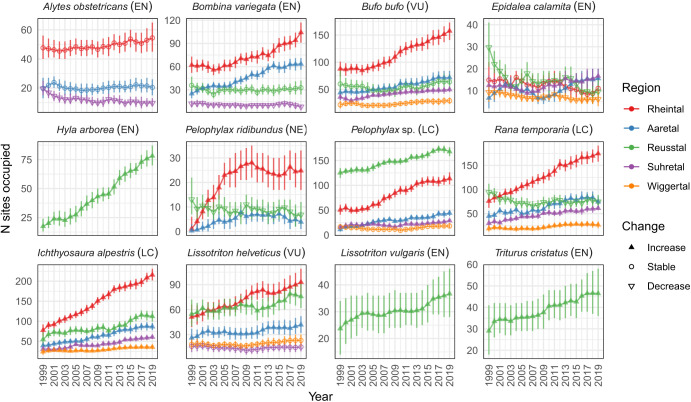
Trajectories of estimated metapopulation size (number of occupied sites; mean with 95% CI) in five regions from 1999 to 2019. Not all species occur in all regions. Filled upward triangles indicate increases in metapopulation size (with certainty >90%), open circles no change, and open downward triangles decreases between 1999 and 2019. EN, VU, NT, LC, and NE in parentheses indicate the national Red List status following IUCN criteria and categories.

Trends in metapopulation sizes differed substantially between regions (Fig. 1). Across all species, the majority (65%) of the 43 regional metapopulations increased in size between 1999 and 2019, 21% remained stable, and 14% declined. Considering only the 25 metapopulations of the eight Red-Listed species, 52% increased, 32% remained stable, while 16% declined. The only species that consistently increased in all regions was the common and widespread *Ichthyosaura alpestris. E. calamita* showed the most variation between regions, with two declining, one stable, and two increasing metapopulations.

There were thus some discrepancies between population change assessed at different spatial scales. The state-wide decline of *E. calamita* masked increasing trends in two regions (Fig. 1), even if these increases were small in absolute terms. Analogously, *A. obstetricans* has been declining at the national scale for decades (27) but has remained stable since 1999 in our study area. To assess progress toward large-scale conservation goals, national-, continental-, and even global-scale analyses are needed (9, 29, 37), but in order to evaluate conservation actions and their success, effects should be considered at the spatial scale where actions took place and are expected to have impact. Pond-breeding amphibians can maintain stable metapopulation sizes despite local extinctions if distances between ponds are shorter than typical dispersal distances. The relevant scale for long-term population management is therefore the metapopulation scale ((38–40); herein operationally defined as the regions).

### Regional Changes in Metapopulation Size Reflect Conservation Effort.

Increases in metapopulation size were most common in Rheintal (seven out of nine species occurring there increased) and Aaretal (eight out of nine species; *SI Appendix*, Fig. S4). These are the regions where the most ponds were constructed (198 and 98 new ponds added to 131 and 72 pre-existing ponds in Rheintal and Aaretal, respectively). Increases were rarest and weakest in Wiggertal, where construction did not begin until 2009 and only 11 ponds were built in addition to 33 existing ponds. Here, only the two common species *Rana temporaria* and *I. alpestris* increased. Second was Suhretal, where 36 ponds were added to 49 existing ponds, beginning in 2006. Three common species and two Red-Listed anurans (notably *E. calamita*) increased there, but two other Red-Listed anurans decreased (Fig. 1). Reusstal, which had the most old ponds (149), showed idiosyncratic patterns: all newts increased, while among seven anurans, three declined, two remained stable, and two increased. The latter included the spectacular recovery of *Hyla arborea*, which showed a nearly fivefold increase in metapopulation size, from 16 occupied sites in 1999 (95% CI: 12, 21) to 77 (95% CI: 70, 86) sites in 2019 (Fig. 1). Even though 79 new ponds were constructed in Reusstal, given the large initial number of ponds, the relative change in habitat availability was comparatively low. In summary, in regions where more new ponds have been built, more metapopulations increased and fewer declined (*SI Appendix*, Fig. S4).

### Newly Constructed Ponds Contributed to Metapopulation Stabilization and Recovery.

**Fig. 2.**
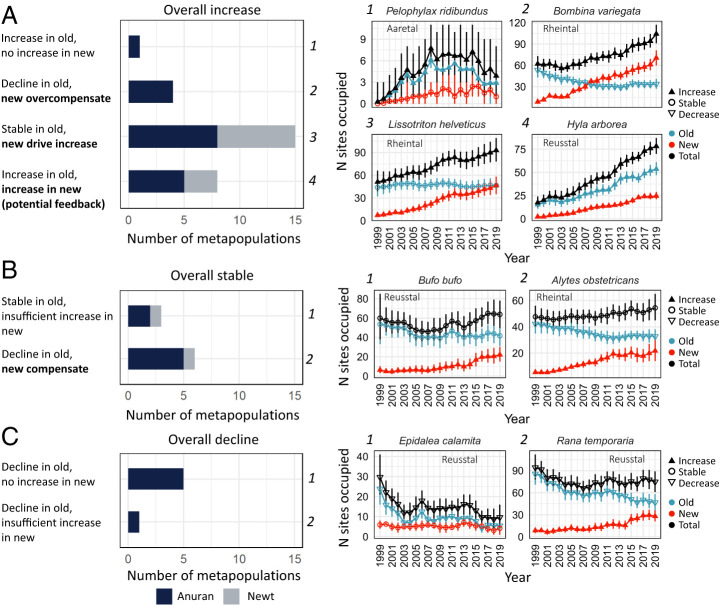
Patterns of change in old and new ponds for 43 regional metapopulations with (*A*) increasing (*n* = 28 metapopulations), (*B*) stable (*n* = 9), and (*C*) overall declining (*n* = 6) metapopulation size from 1999 to 2019 (>90% probability). *Left* panels show the number of metapopulations that follow each of eight possible patterns. Bold font indicates patterns where the colonization of new ponds influenced the overall trend (33 or 77% of the 43 metapopulations). *Right* panels show one example per pattern of a corresponding trajectory of estimated total regional metapopulation size (black), split into the number of old (blue) and new (red) sites occupied. Symbols indicate increasing (filled upward triangles), stable (open circles), or decreasing (open downward triangles) Nos. of occupied ponds from 1999 to 2019 (with >90% probability). Error bars show 95% CI.

To further quantify the role of new ponds in changing metapopulation sizes, we split trajectories of regional metapopulation size into occupied old and new ponds and categorized changes therein based on differences between 1999 and 2019 (with >90% probability; *SI Appendix*, Table S3). Each metapopulation was assigned a pattern of change given the combination of its overall change with changes in old and new ponds (Fig. 2).

The contributions of new ponds to metapopulation recovery followed different patterns. Overall increases in metapopulation size (Fig. 2*A*) were always associated with the colonization of and persistence in new ponds, with the exception of *Pelophylax ridibundus* in Aaretal (Fig. 2*A*.1). This invasive species was present in Reusstal since 1970 but only started invading Aaretal and Rheintal in the late 1990s, where it occupied more old than new ponds (*SI Appendix*, Fig. S5). In all other increasing metapopulations, the occupation of new ponds caused an increase in total metapopulation size, either by overcompensation of declines in old ponds, by directly driving increases, or via concurrent increases in new and old ponds.

Overall stable metapopulation sizes (Fig. 2*B*) were associated either with stable Nos. of occupied old ponds or with the compensation of declines in old ponds by increasing Nos. of occupied new ponds. Overall declines (Fig. 2*C*) occurred where new ponds did not get colonized or not in sufficient Nos. to compensate for declines in old ponds.

Three nonmutually exclusive mechanisms are likely to be involved in the contributions of new ponds to changes in metapopulation sizes. First, the sheer number of new ponds increases the availability of breeding habitat for many species, of which highly mobile generalist species, such as *Bufo bufo*, *I. alpestris*, or *Pelophylax* frogs, should benefit most immediately. These common generalists were not the main conservation targets but profited as a side effect. Species with more-specific habitat requirements should also benefit, as long as structural and ecological variation in constructed ponds includes some suitable habitat for them as well. *E. calamita*, for example, prefers large shallow ponds with fluctuating water tables in open areas (41), while *A. obstetricans* needs terrestrial microhabitats, such as dry stone walls in the surroundings of ponds (23). An insufficient number of new ponds meeting such criteria might explain why these habitat specialists responded less to pond construction. Second, the constant addition of new, early successional ponds to some degree restores the dynamics of natural, undisturbed floodplains or temporarily flooded meadows and marshes. This should benefit pioneer species, such as *Bombina variegata* and *E. calamita*, that preferentially colonize ponds with short hydroperiods devoid of vegetation and predators (41). Successional changes likely explain declines of these species in old ponds (*SI Appendix*, Fig. S5; (41, 42)). This beneficial aspect of pond construction can therefore only be maintained if new ponds are constructed continuously or if active management periodically resets some ponds to early successional stages, which is already practiced (43). Third, the increasing density of and hence connectivity between ponds should benefit dispersal-limited species, such as newts, which rarely disperse farther than a few hundred meters (44). In mobile or already widespread species, increased habitat availability and increased connectivity could jointly cause a positive feedback, leading to higher occupancy of old and new ponds, as was potentially the case for *H. arborea*. The invasive *P. ridibundus* colonized new ponds readily (Fig. 2 and *SI Appendix*, Fig. S5); hence, it was also a beneficiary of pond construction. Similar to American bullfrogs (45), this large frog has negative impacts on native amphibians through predation or competition (46, 47). Nonetheless, most native species showed increased metapopulation sizes in response to pond construction, but the increase might have been even stronger in the absence of invasive species.

### Higher Colonization Rates but Lower Persistence in New Compared with Old Ponds.

**Fig. 3.**
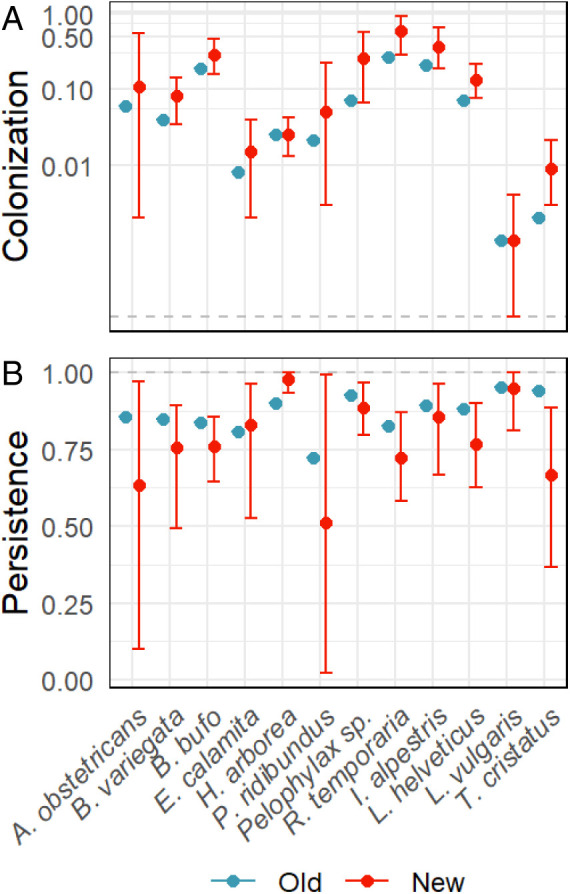
Probabilities of (*A*) colonization and (*B*) persistence in new ponds (red, with 95% CI) compared with mean probabilities in old ponds (blue, point estimates as baseline without associated uncertainty) across all regions. Note the log scale y axis for colonization probability. Estimated probabilities in old and new ponds in individual regions are shown in *SI Appendix*, Fig. S6.

The dynamics of colonization and persistence differed between old and new ponds (Fig. 3 and *SI Appendix*, Fig. S6). Colonization probabilities tended to be higher in new than in old ponds, especially in common species (*Pelophylax* sp., *R. temporaria*, and *I. alpestris*) but also for the rarer *B. variegata*, *Lissotriton helveticus*, or *Triturus cristatus*. This is expected, as new ponds start empty while suitable old ponds may already be occupied. New ponds also differ from old ponds in that they initially lack vegetation and predators and are thereby more attractive to early successional species, such as *B. variegata.*

In contrast, persistence probabilities were lower in new ponds than in old ponds for the majority of species (Fig. 3). Immigrants may not always be able to establish persistent populations. Small initial population sizes render newly established populations prone to local extinction, be it due to Allee effects, local inbreeding, or environmental or demographic stochasticity (48). Lower persistence probabilities can also be expected for early successional species that become locally extinct, once vegetation becomes denser and populations of tadpole predators increase (e.g., *B. variegata*). *H. arborea* was the only species with higher persistence probability in new than in old ponds, which likely contributed to the exceptional increase in metapopulation size of this species.

### Effects of Local and Landscape Variables on Colonization Probabilities.

**Fig. 4.**
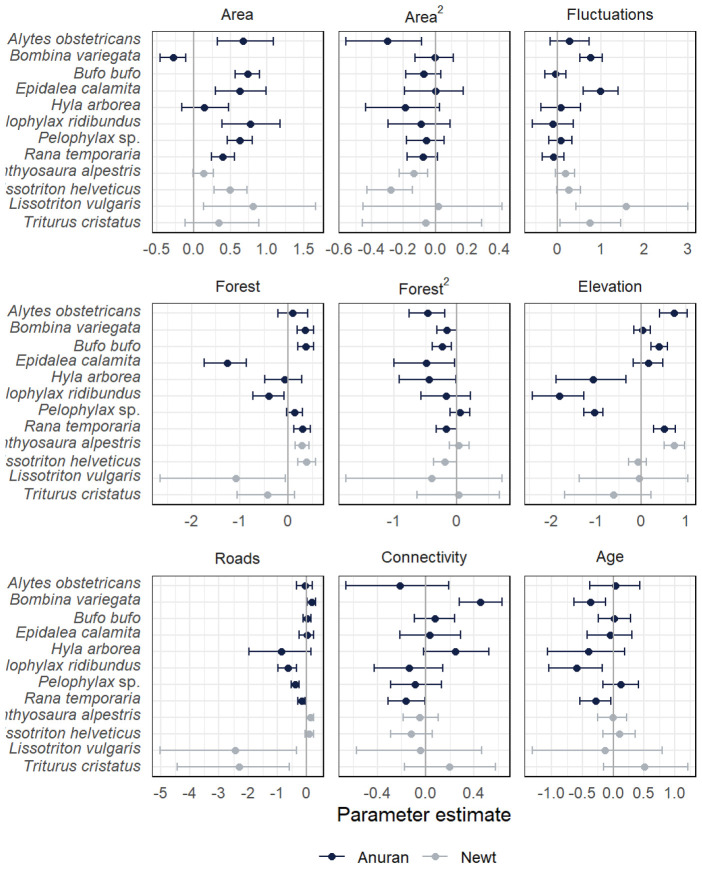
Estimates (logit scale) of the effects of environmental variables on colonization probability (mean, 95% CI). Environmental variables were pond surface area, fluctuations of the water table, the percent area of forest within 100 m of the pond, elevation, the area of large (width ≥6 m) roads within 1 km of the pond, connectivity, and the age of newly constructed ponds. Absolute values of parameter estimates indicate effect size, as all covariates were standardized to mean zero and unit variance prior to analysis.

To inform future conservation practice (23), we assessed the influence of the age of ponds and of environmental variables describing the ponds and the surrounding landscape on colonization probabilities. Colonization rates depended on both pond and landscape characteristics, suggesting complementary effects of both aquatic and terrestrial habitat for completing the complex amphibian life cycle ((44, 49); Fig. 4 and *SI Appendix*, Table S4). Colonization probability increased with pond surface area continuously or unimodally for all species but *B. variegata*, which had a higher colonization probability in smaller ponds. Water-table fluctuations, indicating variation in pond hydroperiod length and the potential for pond drying, increased colonization probabilities of *B. variegata* and *E. calamita*, as well as all newt species. This constitutes important information for practitioners, as both the surface area of ponds and water-table fluctuations can be specified during pond construction (23, 50). In our study landscape, for example, only 3% of newly constructed ponds had a surface area >1,000 m^2^ and fluctuating water tables. A lack of large ponds with variable hydroperiod lengths may be a limiting factor for the recovery of *E. calamita* in this landscape.

Landscape complementation is important when selecting locations for new ponds, as amphibians require both suitable aquatic habitat for larvae and terrestrial habitat for adults (23, 49). *Epidalea calamita*, *P. ridibundus*, and *Lissotriton vulgaris* had higher colonization probabilities in ponds with less forest cover in the surroundings, while most other species showed unimodal or positive responses to forest cover (Fig. 4). Roads can have negative effects on dispersing or migrating amphibians (44). Here, we found negative effects of roads on colonization probabilities that were significant for the frog species and the strongly affected newts *L. vulgaris* and *T. cristatus*, which generally had low colonization probabilities (Fig. 4). Structural connectivity, integrating the density and vicinity of other ponds, increased colonization probability of *B. variegata*, *H. arborea*, and, to some extent, *T. cristatus*, while *R. temporaria* more frequently colonized more isolated ponds. Pond age distinguished the early and fast colonizers (*B. variegata*, *P. ridibundus*, and *R. temporaria*) from species preferring late successional stages (*T. cristatus*).

Summarizing landscape effects at the most general level, our results show that the rapid colonization of new amphibian breeding ponds is aided by constructing them close to forest, far from roads, and in proximity to other ponds. However, some species showed idiosyncratic responses, and for most species, the effects of landscape variables and structural connectivity were relatively weak. This supports the encouraging conclusion that no effort in pond construction is really wasted. Some species will benefit, and diverse landscape settings are ultimately required to maintain a heterogeneous community of species.

### Pond Construction as a Conservation Tool for Amphibians: Lessons Learned.

Large-scale and long-term surveys provide invaluable information on population trends (7, 51) and their drivers (9, 52), but few monitoring programs link population trends directly to specific conservation measures (but see refs. (8, 14)). We had the rare opportunity to analyze data from a monitoring program that accompanied 20 y of habitat creation, showing that the landscape-scale construction of new ponds halted or reversed the decline of most pond-breeding amphibian species in a human-dominated landscape. This success was achieved with a relatively simple but massive and persistent conservation program. Crucially, this landscape-scale recovery of the majority of species took place despite ongoing pressures from other stressors of amphibians, such as increasing urbanization, a high density of roads with high traffic volumes, the presence of nonnative fish and pathogens, and/or pesticide use. Similarly, the removal of nonnative fish was sufficient to induce population recovery of a pond-breeding amphibian in Yosemite National Park, despite the continued threat from chytrid fungus (14). Addressing individual drivers of population decline, here countering habitat loss through pond creation, can make a difference.

The Swiss lowlands are similar to large parts of temperate Europe and North America, where—as a legacy of Pleistocene glaciations—communities of amphibians are species poor with pond breeding as the dominant reproductive strategy and where human impacts are pervasive and severe. Habitat loss was a key driver of species declines (37), and pond construction can therefore be an effective tool for amphibian conservation in such landscapes (22). While this is good news for amphibian conservation, it also points to an important limitation of our study: the findings may not be generalizable to other parts of the world, e.g., the tropics, where amphibian communities are very diverse and where other reproductive strategies predominate (terrestrial breeding, stream breeding, or the use of phytotelmata). Other conservation strategies are necessary in such places. Another point to consider is that, despite a high human population density and dense traffic infrastructure, the state Aargau with 37% forest cover may be more permeable to amphibian migration than other landscapes. New ponds may not get colonized as readily in intensively farmed agricultural land with large-scale fields and no natural landscape elements.

Pond construction on agricultural land does take place on a massive scale in many parts of the world, albeit not for amphibians but for watering livestock. If properly managed, farm ponds can constitute important breeding habitats for endangered amphibians (53, 54), especially if different successional stages are available, cattle grazing is not too intense, and if there are no fish (55). If cattle ponds are managed in a biodiversity-friendly way, they may contribute to the recovery of amphibians and freshwater biodiversity (56). This is an important lesson also from the present study: ensuring habitat heterogeneity by providing a diverse set of ponds with different sizes, hydroperiods, and terrestrial habitats will spread the benefits of pond construction across many species, as long as source populations exist within their dispersal distance (57). At the scale of the entire study area, only two species with very specific habitat requirements did not benefit from pond construction. Promoting *E. calamita* would likely require the construction of more large and shallow ponds with short hydroperiods (50), and *A. obstetricans* could be supported by increasing the availability of suitable sheltering habitats around ponds (23).

The observed recovery of the majority of species is a remarkable and encouraging conservation outcome. It was possible because the responsible authorities committed to a major conservation effort early on and many actors contributed to pond construction. Semlitsch pointed out 2 decades ago (57) that the critical elements required to initiate effective recovery for amphibians were known. Habitat loss remains a key driver of amphibian declines, and the continuous provision of new breeding habitat here was sufficient to bend the curve, despite other negative impacts. The foresight of accompanying the effort with comprehensive monitoring made it possible to document the successes and learn from the (few) failures, facilitating evidence-based amphibian conservation (5).

## Materials and Methods

### Study Area, Species, and Monitoring Data.

The Swiss state Aargau has a total area of 1,404 km^2^, of which 46% is in agricultural use, 37% is forest, and 15% is urban area; it covers an altitudinal range from 260 m to 908 m above sea level. It is densely populated, intensively used, and highly fragmented by urban areas and traffic infrastructure (*SI Appendix*, Fig. S1). We used monitoring data for all 12 extant pond-breeding amphibian species (*SI Appendix* S1).

The monitoring is focused on five regions with significant remnant populations of threatened amphibian species ((30); *SI Appendix*, Fig. S2). All breeding sites potentially suitable for a target species (*SI Appendix*, Table S1) were surveyed comprehensively and regularly in a rotating panel design. Because the regions were large, they were subdivided into subregions for annual surveys. Between 1999 and 2019, each subregion was surveyed on average 5.5 times with a mean (± SD) period of 3.8 (±1.38) y between surveys.

Over the last decades, multiple actors (state authorities, nonprofit organizations, and private landowners) have together constructed hundreds of new ponds to benefit threatened amphibians. Estimated pond construction costs are given in *SI Appendix* S2. Within the study regions, the total number of sites increased from 469 in 1999 to 780 in 2019 through the construction of new habitats (*SI Appendix*, Fig. S2). Four hundred twenty-two sites were added, while 76 sites were destroyed through drainage or infill.

A total of 856 sites entered the analysis, 434 of which were old sites and 422 were new sites, i.e., constructed since 1991. For 362 of the new sites, the exact year of construction could be determined (from information from the agencies that built the sites or from aerial photographs). Of these, 35 were constructed during the 1990s, all others after 1999. For 60 new sites, the exact year of construction was unknown. Where there was uncertainty in the year of construction or destruction, we conservatively assumed the minimum duration of existence, i.e., sites were assumed to exist from the first or until the last year, respectively, in which there was survey data for them and assumed to be nonexistent before or after these years.

Within a survey year, each site of a surveyed subregion was visited on average 2.8 times (SD 0.5). The first two visits occurred at night and the third during day (mainly intended to detect diurnal species and tadpoles). Over the more than 20 y of monitoring, a total of 260 trained volunteers, organized by a professional coordination office, conducted the fieldwork using a standardized field protocol (*SI Appendix* S3). During each visit, the presence of species was recorded based on detection of any life stage or of calls. Not all species occurred in all regions (*SI Appendix*, Table S1). Regions with ≤10 sites with detections were excluded from modeling for that species.

### Covariate Data.

We included pond and landscape characteristics that could affect colonization probabilities in the analysis (*SI Appendix*, Fig. S7). Characteristics of the sites were elevation (meters above sea level), surface area of all water bodies at a site (m^2^, log-transformed), and fluctuations of the water table (0/1), all recorded in the monitoring scheme, as well as the age (years since construction) of newly constructed ponds. The surroundings of each site were characterized by the percent area of forest within a circular buffer of radius 100 m and the area of large (width ≥6 m) roads within a circular buffer of radius 1 km, extracted from the swissTLM3D vector data (swissTLM3D, SwissTopo [5704000000]) in QGIS v.3.16 (https://qgis.org/en/site/). We calculated these two variables at different radii because we expected them to affect different aspects of species’ ecology. Forest cover was assumed to be relevant as terrestrial habitat (landscape complementation sensu (23, 49)). Roads were assumed to affect long-distance seasonal migrations and dispersal between ponds (44, 58). We included a metric of structural connectivity derived from metapopulation theory (59), calculated as $C_{it}=\;\sum_{j\neq i}\text{exp}(-d_{ij})$, where $d_{ij}$ is the pairwise Euclidean distance (km) between all sites existing in year *t*. This metric assumes a negative exponential dispersal kernel with mean dispersal distance of 1 km. The difference between new and old ponds was included as a categorical effect for both colonization and persistence probabilities. All continuous covariates were centered and scaled to mean zero and unit variance before analysis.

### Statistical Analysis.

For each species, we fitted a dynamic occupancy model to species detection–nondetection data at the site level using Bayesian inference (60). The observed data $y_{\mathrm{ijt}}\;$ in site *i*, visit *j*, and year *t* are related to the true latent occupancy state $z_{it}$ via detection probability as $y_{\mathrm{ijt}}∼\mathrm{Bernoulli}(z_{it}p_{\mathrm{ijt}}$). Detection probability was estimated from repeat visits to the same site during each survey year. Observer identity was added as a random effect for nighttime surveys. Additionally, we distinguished between detection probability during nighttime visits (j∈{1,2}) and the daytime visit (j=3), such that detection probability was estimated from the three repeat visits *j* as

$$\mathrm{logit}\left(p_{\mathrm{ijt}}\right)=\{\begin{array}{c} \alpha_{p}+\;\beta_{\mathrm{Obs}(\mathrm{ijt})},\;j\in \left\{1,2\right\}\\ \alpha_{p}+\;\beta_{\mathrm{day}},\;j=3 \end{array},$$

where $\beta_{\mathrm{Obs}(\mathrm{ijt})}∼N(0,\;\sigma_{\mathrm{Obs}}^{2}$) (results in *SI Appendix*, Table S5).

Initial occupancy probability in the first year was estimated for all existing sites *i* ($E_{i1}=1$) as a region-specific intercept: $z_{i1}∼\mathrm{Bernoulli}(\mathrm{logit}(\alpha_{\psi 1,\mathrm{Region}})E_{i1}$), with $\alpha_{\psi 1,\mathrm{Region}}∼N(\mu_{\psi 1},\;\sigma_{\psi 1}^{2}$). Occupancy probability in all subsequent years was modeled, for all existing sites, as a function of colonization probability $\gamma_{it}$ and persistence probability $\varphi_{i}$, as $z_{it}∼\mathrm{Bernoulli}(\left[\left(1-z_{i,t-1}\right)\;\gamma_{it}+z_{i,t-1}\;\varphi_{i}\right]\;E_{it}$). Our main interest was the colonization process. We therefore modeled colonization probability as a function of all available covariates, with a region-specific intercept and region-specific difference of new as compared with old ponds, as

$$\mathrm{logit}(\gamma_{it})=\alpha_{\gamma ,\mathrm{Region}(i)}+\beta_{\mathrm{New}(i),\;\mathrm{Region}(i)}+\underset{k}{\sum }\beta_{k}X_{\mathrm{kit}}$$

for all *k* covariates listed above, with $\alpha_{\gamma ,\mathrm{Region}}∼N(\mu_{\alpha ,\gamma },\;\sigma_{\alpha ,\gamma }^{2}$) and $\beta_{\mathrm{New},\mathrm{Region}}∼N(\mu_{\beta \mathrm{New}},\;\sigma_{\beta \mathrm{New}}^{2}$). Persistence probability was modeled with a region-specific intercept and region-specific difference of new as compared with old ponds, as

$$\mathrm{logit}(\varphi_{i})=\alpha_{\varphi ,\mathrm{Region}(i)}+\delta_{\mathrm{New}(i),\;\mathrm{Region}(i)}$$

with $\alpha_{\varphi ,\mathrm{Region}}∼N(\mu_{\alpha ,\varphi },\;\sigma_{\alpha ,\varphi }^{2}$) and $\delta_{\mathrm{New},\mathrm{Region}}∼N(\mu_{\delta \mathrm{New}},\;\sigma_{\delta \mathrm{New}}^{2}$).

Vague priors were assumed for all parameters: probabilities were given Uniform(0,1) priors on the probability scale, SDs Uniform(0,10) and linear slope estimates Uniform(−10,10) priors on the logit scale. For each model, we estimated posterior distributions using three Markov chain Monte Carlo chains, each with a discarded burn-in of 30,000 iterations and subsequent 100,000 iterations thinned by 250, resulting in posterior samples of size 1,200 on which inference was based. Convergence was assessed by visual inspection and via the Brooks–Gelman–Rubin statistic (60). Goodness of fit was assessed using posterior predictive checks (60), comparing the observed number of occupied sites during core area survey years with predictions under the model (*SI Appendix*, Fig. S8). Models were fitted in JAGS, run through R version 4.0.3 with package jagsUI (code in *SI Appendix* S5).

## Supplementary Material

Supplementary File

## Data Availability

Anonymized [Species detection/nondetection data for 12 amphibian species from 1999 to 2019 in 856 ponds along with covariate data describing ponds and surroundings of ponds.] data and code have been deposited in [EnviDat] (https://www.doi.org/10.16904/envidat.270) (61).
