# Supplementary material for: Bending the curve: Simple but massive conservation action leads to landscape-scale recovery of amphibians
Source: Proc Natl Acad Sci U S A. 2022 Oct 10;119(42):e2123070119. doi: 10.1073/pnas.2123070119 (PMC9586276; doi:10.1073/pnas.2123070119)
Supplement: Supplementary File [file pnas.2123070119.sapp.pdf]

PNAS
www.pnas.org

Supplementary Information for
Bending the curve: Simple but massive conservation action leads to landscape-scale recovery of amphibians

Helen Moor, Ariel Bergamini, Christoph Vorburger, Rolf Holderegger, Christoph Bühler, Simon Egger, Benedikt R. Schmidt

Helen Moor
Email: helen.moor@wsl.ch

This PDF file includes:

Supplementary text
Figures S1 to S8
Tables S1 to S5
SI References

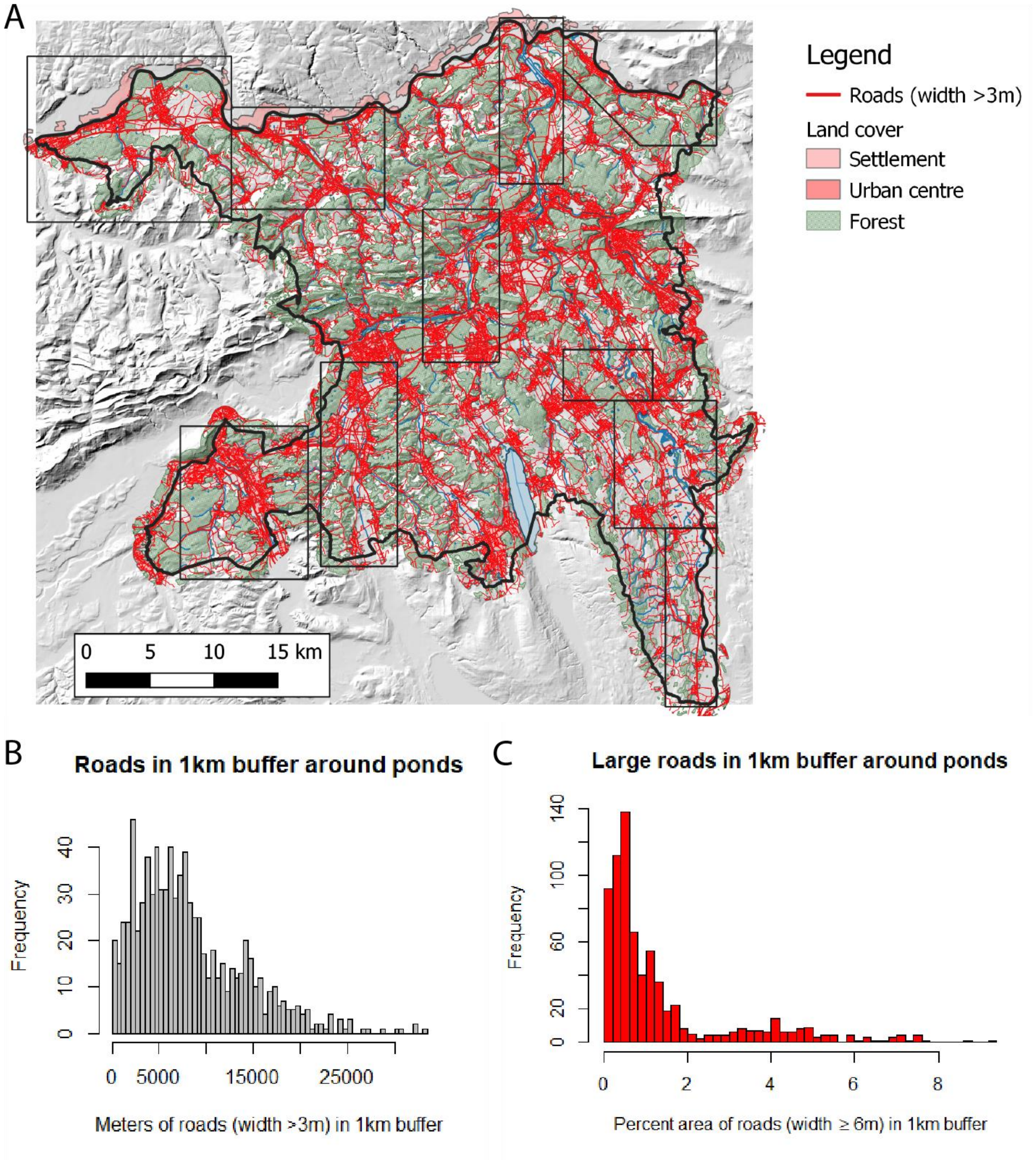

Fig. S1. (A) Map of the state Aargau showing the dense network of urban areas and roads, interspersed with forest patches and agricultural land. (B) Histogram of the length of roads wider than 3m within a radius of 1km around the studied ponds. All ponds have some roads (width >3m) in their surroundings. (C) Histogram of the percent area that is covered by large roads (width ≥6m) within a radius of 1km around the studied ponds. All ponds have some large roads in their surroundings; for 18% of ponds, more than 2% of the surrounding land surface is covered by large roads. In the analysis, we considered large roads (width ≥6m).

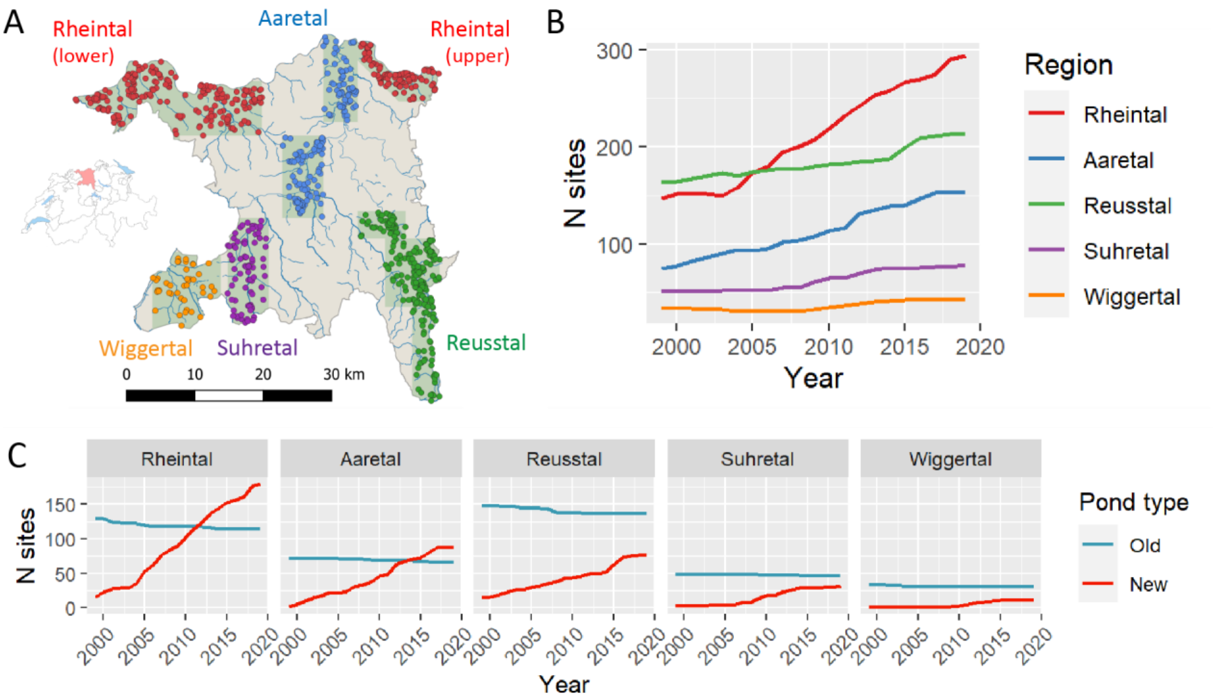

Fig. S2. (A) Map of the state Aargau showing all 856 ponds studied, as distributed across the five regions (indicated by color). Light green boxes show the subregions of the survey design which were aggregated for the analyses to regions following major river valleys. (B) Number of existing sites from 1999 to 2019 in the five regions. (C) Number of old (existing before 1991) and new (created since 1991) ponds in the five regions. Old ponds can get lost over time, e.g., through infilling or overgrowth. In Rheintal, most ponds have been built; in Suhretal and Wiggertal, pond construction started later and fewer new ponds were created.

SI Appendix S1. Ecology and natural history of amphibians in the study area.

The amphibian species in the study area (Swiss state Aargau) differ strongly in their ecology, habitat requirements and natural history. Here we summarize some key ecological characteristics of the species.

Alytes obstetricans. Average total length is 47 mm (1). This species was Red-Listed as endangered (EN) because of population declines (2). The species mates on land and the male takes care of the clutch (3). The larval period last usually more than one year (4); size at metamorphosis is relatively large (~40% of adult size) (1). Sexual maturity is reached on average after 2-3 years (1). The species therefore prefers permanent ponds without fish (5). This species is negatively affected by invasive *Pelophylax* frogs (6) and the pathogenic amphibian chytrid fungus (7). Connectivity was found to predict pond occupancy (8). Dispersal distance is less than 700 m (9).

Bombina variegata. Average total length is 44 mm (1). Sexual maturity is reached on average after 1-2 years (1). It is Red-Listed as endangered (EN) because of population declines (2). The species prefers small temporary ponds and is often found in ponds without competitors (often less than a square meter; (10, 11)). It is negatively affected by invasive *Pelophylax* frogs (6) and prevalence of the amphibian chytrid fungus is high (7). Clutch size is small, reproduction and adult survival varies among populations and depends on the stability of the habitat (12). Dispersal depends strongly on the predictability of the habitat (13). It can disperse several kilometers (9).

Bufo bufo. Average total length is 77 mm (1). Sexual maturity is reached on average after three years (1). The species is Red-Listed as vulnerable (VU, (2)) because population declines were observed (14). Prefers deep, permanent but warm ponds, often with fish (15–17). The terrestrial habitat used during the summer can be several kilometers away from the breeding site (3). Observed dispersal distances are 3-4 km. Connectivity was found to predict pond occupancy (8, 18).

Epidalea calamita. Average total length is 63 mm (1). Sexual maturity is reached on average after three years (1). It is Red-Listed as endangered (EN) because of population declines (EN, (2)). Clutch size is large (4000 eggs). Larval development can be completed in less than a month. It prefers large temporary ponds, mostly very ephemeral pools (5). Small populations have high extinction rates (19). This species is negatively affected by invasive *Pelophylax* frogs (6). It can disperse several kilometers; distance appears to depend on the soil type (20).

Hyla arborea. Average total length is 42 mm (1). It is Red-Listed as endangered (EN) because of population declines (2). Small populations have high extinction rates (19, 21). Sexual maturity is reached on average after one year (1). Annual survival is low (~30%; (22)). The species prefers sunny temporary ponds (5, 15). Connectivity was found to predict pond occupancy (18).

Pelophylax sp. Average total length is 88 mm (1). Sexual maturity is reached on average after three years (1). The two native *Pelophylax* frogs (*P. lessonae* and *P. esculentus*) form a hybridogenetic hybrid complex (23). Several species of non-native *Pelophylax* frogs occur in Switzerland, notably *P. ridibundus* (24, 25). *Pelophylax* frogs are not on the Swiss national Red List and are commonly found in lentic water bodies, primarily in permanent ponds (5) but there are subtle differences in habitat preferences between the native species related to the predator community. *Pelophylax esculentus* is often found with fish but relative abundance is lower in ponds with invertebrate predators where *P. lessonae* is more abundant (26). *Pelophylax* frogs can be hard to distinguish based on external morphology (3), hence we grouped *P. esculentus* and *P. lessonae* in our analyses. In the presence of *Pelophylax* frogs, the abundances of *Epidalea calamita*, *Bombina variegata* and *Alytes obstetricans* are lower than in their absence (6). The maximum recorded dispersal distance is 15 km but distances of 1 to 3.5 km are more common (9). Connectivity was found to predict pond occupancy (8, 18).

Rana temporaria. Average total length is 70 mm (1). Sexual maturity is reached on average after three years (1). It is the most common anuran in Switzerland (27). Occurs in a wide variety of ponds, but prefers shallow ponds in areas with high forest cover (15, 17); results on the preference for large and small ponds are inconsistent (15, 17). Pond occupancy is low in areas with high road density (15, 18). Adults are thought to disperse up to 2 km (9). Connectivity was found to predict pond occupancy (18).

Ichthyosaura alpestris. Average total length is 95 mm (1). Sexual maturity is reached on average after three years (1). The Alpine newt is the most common newt in Switzerland (3, 27). It is not threatened

in Switzerland (28). It can be found in almost all types of lentic water bodies, even sometimes in those containing fish, albeit low abundance. It is also commonly found in ponds in urban areas. Luqman et al. (29) used genetic data to show that the landscape poses no important obstacles to dispersal between sites but Van Buskirk (16) found that urban areas and transportation infrastructure impede dispersal. Adults were found to disperse up to 4 km (9). Connectivity was found to predict pond occupancy (18).

Lissotriton helveticus. Average total length is 76 mm (1). Sexual maturity is reached on average after two years (1). This newt is widespread in the Swiss lowlands and listed as vulnerable (VU) on the national Red List. It occurs in a wide variety of pond types but prefers rather shady ponds near forests (30). Adults were found to disperse up to 800 m (9). Connectivity was found to predict pond occupancy (8).

Lissotriton vulgaris. Average total length is 84 mm (1). Sexual maturity is reached on average after 2-3 years (1). This species is the rarest newt in Switzerland and ranked as endangered (EN) on the national Red List because of strong population declines (2). It is most commonly found in medium-sized ponds in the alluvial zones along the rivers but tends to avoid forests (15). Adults were found to disperse up to 1.275 km (9). Connectivity was found to predict pond occupancy (8).

Triturus cristatus. Average total length is 140 mm (1). Sexual maturity is reached on average after 2-3 years (1). This rare newt species has undergone strong population declines and a stark reduction of the distribution range (2, 27) and is therefore listed as endangered (EN) on the national Red List. It prefers ponds that dry infrequently and have well developed vegetation of submersed macrophytes in open landscapes (15, 31). It rarely disperses further than 500 meters and preferentially to ponds with high habitat suitability (32). Because larvae grow to a large size in a short time, it requires ponds with high zooplankton density (33).

Table S1. Species list, with Red List status in Switzerland in brackets (34). Target species of the pond construction program are marked with an asterisk. N sites gives the total number of old (existing before 1991) and new (newly constructed since 1991) sites in five regions. The number of sites with at least one detection between 1999 and 2019 per species and region, split into old and new sites, is given. Regions with ≤10 sites with detections were excluded from analysis for that species (italicized). Three species (*Hyla arborea*, *Lissotriton vulgaris*, *Triturus cristatus*) had sufficient data for analysis in a single region only (Reusstal). LC: least concern; VU: vulnerable; EN: endangered; NE: not evaluated.

	**Rheintal ($215\ km^2$)**		**Aaretal ($132\ km^2$)**		**Reusstal ($146\ km^2$)**		**Suhretal ($84\ km^2$)**		**Wiggertal ($82\ km^2$)**	
	Old	New	Old	New	Old	New	Old	New	Old	New
**N sites**	131	198	72	98	149	79	49	36	33	11
**Species** (Red List status)										
Alytes obstetricans (EN)*	47	33	30	9	*9*	*1*	20	3	*4*	*0*
Bombina variegata (EN)*	62	93	41	55	49	28	15	7	*5*	*0*
Bufo bufo (VU)	98	117	61	43	93	26	41	17	28	9
Epidalea calamita (EN)*	14	14	19	9	33	15	18	11	11	0
Hyla arborea (EN)*	*7*	*2*	*6*	*0*	58	24	*0*	*0*	*0*	*0*
Pelophylax sp. (LC)*	68	92	37	29	130	61	26	8	18	7
Pelophylax ridibundus (NE)	22	24	9	6	24	8	*5*	*2*	*0*	*0*
Rana temporaria (LC)	95	146	60	74	116	47	44	28	29	10
Ichthyosaura alpestris (LC)	106	156	60	68	102	54	45	26	29	10
Lissotriton helveticus (VU)	63	54	36	23	82	29	19	7	23	4
Lissotriton vulgaris (EN)*	*5*	*4*	*7*	*1*	33	12	*5*	*0*	*0*	*0*
Triturus cristatus (EN)*	*4*	*3*	*1*	*1*	48	19	*1*	*0*	*0*	*0*

Table S2. Across the entire studied landscape, ten species occupied a greater number of ponds in 2019 as compared to 1999, one species (*Alytes obstetricans*) showed no change, and one species (*Epidalea calamita*) declined (with 93% probability). Post. dens. <0 gives the proportion of the posterior density of the estimated difference between 1999 and 2019 that is below zero. The number of available sites are all ponds in the regions where a species' occurrence was modelled. Given are the estimated number of occupied ponds across all five regions in 1999 and 2019, the difference in the number of occupied ponds in 2019 as compared to 1999 (mean with 95% CI) and the mean difference expressed as percent change relative to 1999.

Species	Number of sites available	Estimated number of occupied sites 1999 (95% CI)	Estimated number of occupied sites 2019 (95% CI)	Difference 2019-1999 (95% CI)	Post. dens. <0	Percent change (relative to 1999)
A. obstetricans	584	86 (75, 99)	84 (73, 99)	-3 (-19, 14)	0.60	-3
B. variegata	812	136 (119, 154)	207 (190, 226)	**71 (46, 97)**	0	52
B. bufo	856	241 (213, 270)	364 (339, 391)	**123 (83, 160)**	0	51
E. calamita	856	72 (59, 89)	59 (49, 68)	*-14 (-31,4)*	0.93	*-19*
H. arborea	228	16 (12, 21)	77 (70, 86)	**61 (52, 70)**	0	382
P. ridibundus	727	14 (8, 22)	35 (25, 45)	**21 (9, 33)**	0	71
Pelophylax sp.	856	217 (201, 234)	371 (354, 391)	**154 (128, 179)**	0	152
R. temporaria	856	253 (224, 279)	395 (372, 422)	**142 (104, 181)**	0	56
I. alpestris	856	221 (194, 243)	504 (484, 530)	**284 (249, 315)**	0	129
L. helveticus	856	160 (135, 189)	234 (210, 260)	**74 (37, 110)**	0	46
L. vulgaris	228	23 (15, 35)	37 (29, 46)	**14 (2, 29)**	0	59
T. cristatus	228	28 (18, 40)	45 (36, 58)	**17 (3, 34)**	0	62

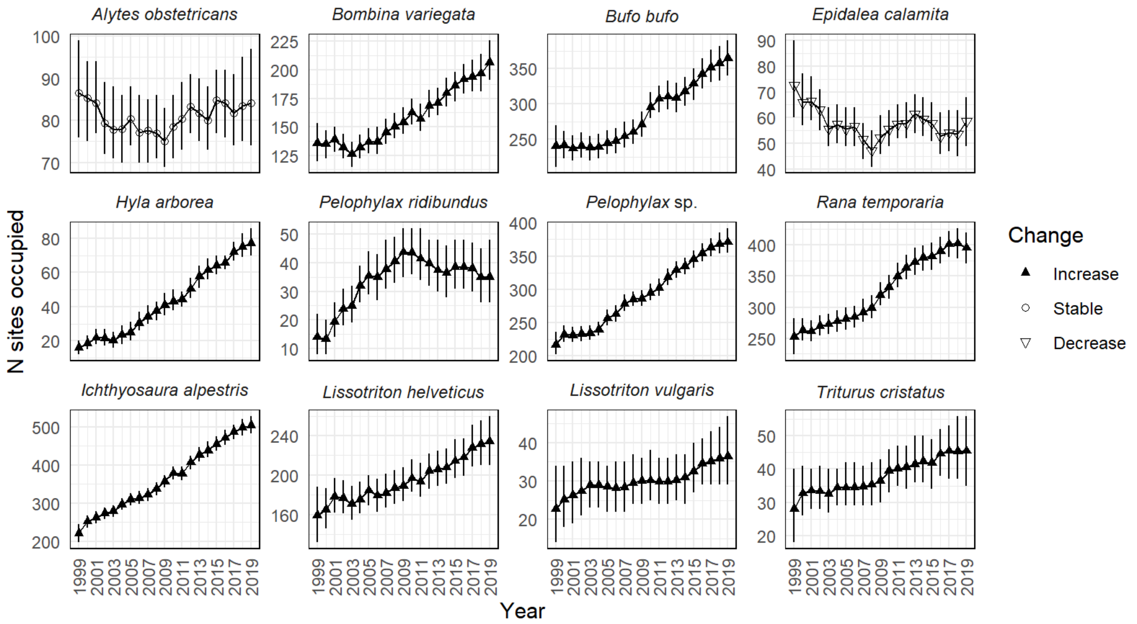

Fig. S3. Trajectories of the estimated number of occupied sites at the landscape scale (across all five regions modelled; mean and 95% CI). Symbols indicate whether the number of occupied sites has increased, remained stable, or decreased in 2019 as compared to 1999 (with >90% probability).

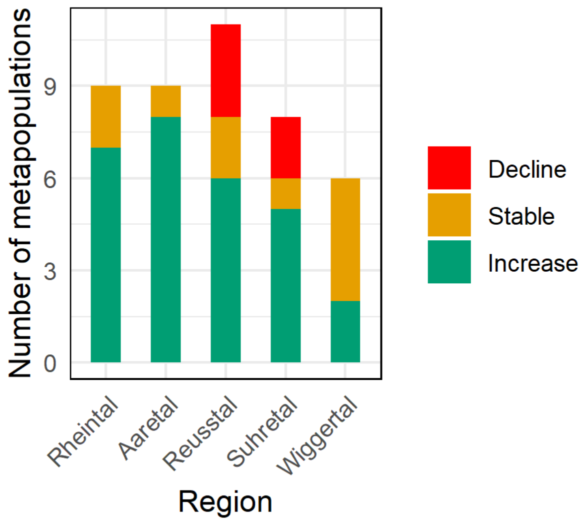

Fig. S4. Number of metapopulations (among 43 regional metapopulations) that are declining, stable, and increasing (with >90% probability) in each of the five regions. Regions are ordered by the number of newly constructed ponds: Rheintal (198) > Aaretal (98) > Reusstal (79) > Suhretal (36) > Wiggertal (11).

Table S3. Patterns of contribution of new ponds to the change in total metapopulation size in five regions: no effect of new ponds; insufficient compensation, compensation, or overcompensation of population declines in old ponds; increase in total metapopulation size due to colonization of new ponds; or double increase in both old and new ponds. Bold font and light grey shading indicate an increase in total metapopulation size; italics and dark grey shading a decrease in total metapopulation size; regular font and no shading correspond to stable metapopulation size. Symbols (–↑↓) indicate >90% probable changes from 1999 to 2019 in the number of occupied old and new ponds, as calculated from the posterior distributions of the differences in numbers of occupied ponds in 2019 as compared to 1999.

**Species**	**Rheintal**		**Aaretal**		**Reusstal**		**Suhretal**		**Wiggertal**	
	Old	New	Old	New	Old	New	Old	New	Old	New
A. obstetricans	Compensation		Compensation				*No effect*			
	↓	↑	↓	↑			↓	–		
B. variegata	**Overcomp.**		**Increase**		Compensation		*No effect*			
	↓	↑	–	↑	↓	↑	↓	–		
B. bufo	**Overcomp.**		**Increase**		No effect		**Increase**		No effect	
	↓	↑	–	↑	–	↑	–	↑	–	↑
E. calamita	Compensation		**Increase**		*No effect*		**Overcomp.**		*No effect*	
	↓	↑	–	↑	↓	–	↓	↑	↓	–
H. arborea					**Double increase**					
					↑	↑				
P. ridibundus	**Double increase**		**No effect**		*No effect*					
	↑	↑	↑	–	↓	–				
Pelophylax sp.	**Increase**		**Double increase**		**Increase**		**Double increase**		Compensation	
	–	↑	↑	↑	–	↑	↑	↑	↓	↑
R. temporaria	**Increase**		**Overcomp.**		*Insufficient comp.*		**Double increase**		Increase	
	–	↑	↓	↑	↓	↑	↑	↑	–	↑
I. alpestris	**Double increase**		**Increase**		**Double increase**		**Double increase**		**Increase**	
	↑	↑	–	↑	↑	↑	↑	↑	–	↑
L. helveticus	**Increase**		**Increase**		**Increase**		Compensation		No effect	
	–	↑	–	↑	–	↑	↓	↑	–	↑
L. vulgaris					**Increase**					
					–	↑				
T. cristatus					**Increase**					
					–	↑				

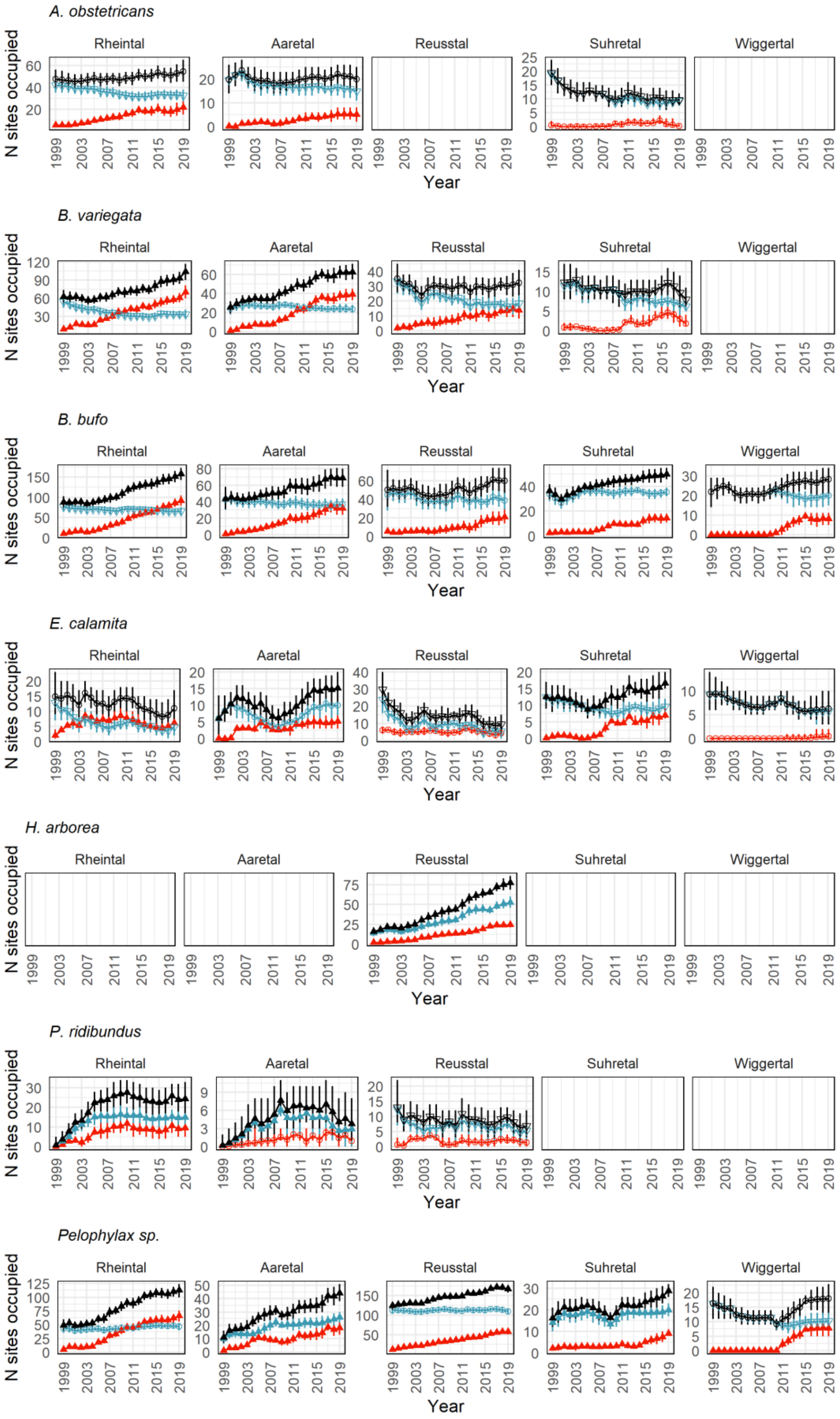
A. obstetricans
Rheintal
Aaretal
Reusstal
Suhretal
Wiggertal
N sites occupied
Year
B. variegata
Rheintal
Aaretal
Reusstal
Suhretal
Wiggertal
N sites occupied
Year
B. bufo
Rheintal
Aaretal
Reusstal
Suhretal
Wiggertal
N sites occupied
Year
E. calamita
Rheintal
Aaretal
Reusstal
Suhretal
Wiggertal
N sites occupied
Year
H. arborea
Rheintal
Aaretal
Reusstal
Suhretal
Wiggertal
N sites occupied
Year
P. ridibundus
Rheintal
Aaretal
Reusstal
Suhretal
Wiggertal
N sites occupied
Year
Pelophylax sp.
Rheintal
Aaretal
Reusstal
Suhretal
Wiggertal
N sites occupied
Year

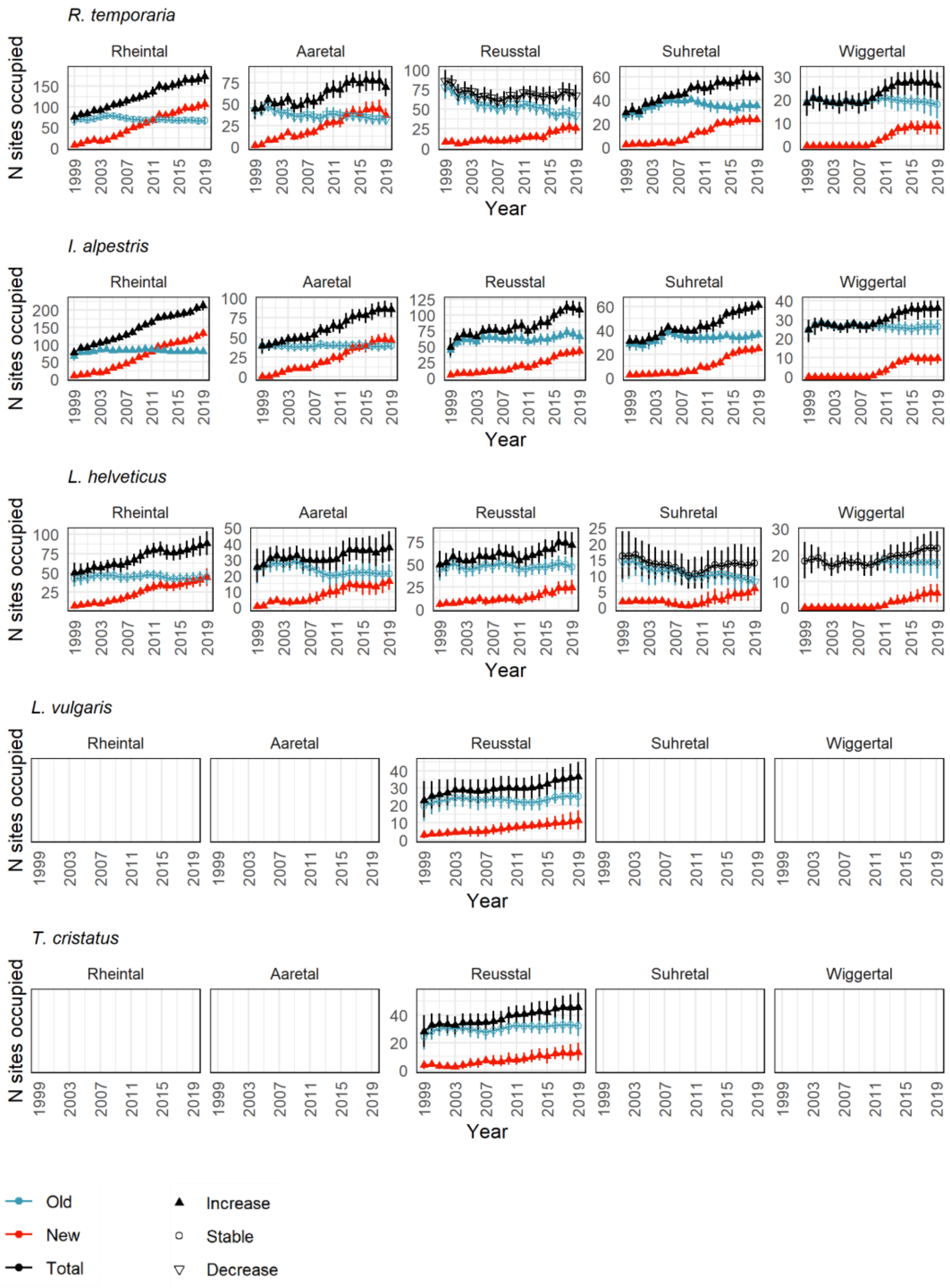

Fig. S5. Metapopulation size (estimated number of occupied ponds, black) in five regions between 1999 and 2019, split into number of occupied old (blue) and newly constructed (red) ponds. Symbols indicate the change in metapopulation size in 2019 as compared to 1999 (classified with >90% probability into increasing, stable, or decreasing size). Empty panels are regions where this species did not occur in sufficient numbers for modelling. *Pelophylax ridibundus* is an invasive species that had been present in Reusstal since the 1970s, but began invading Rheintal and Aaretal in the late 1990s only (25); the trajectory shown here effectively traces its invasion of Rheintal and Aaretal since 1999.

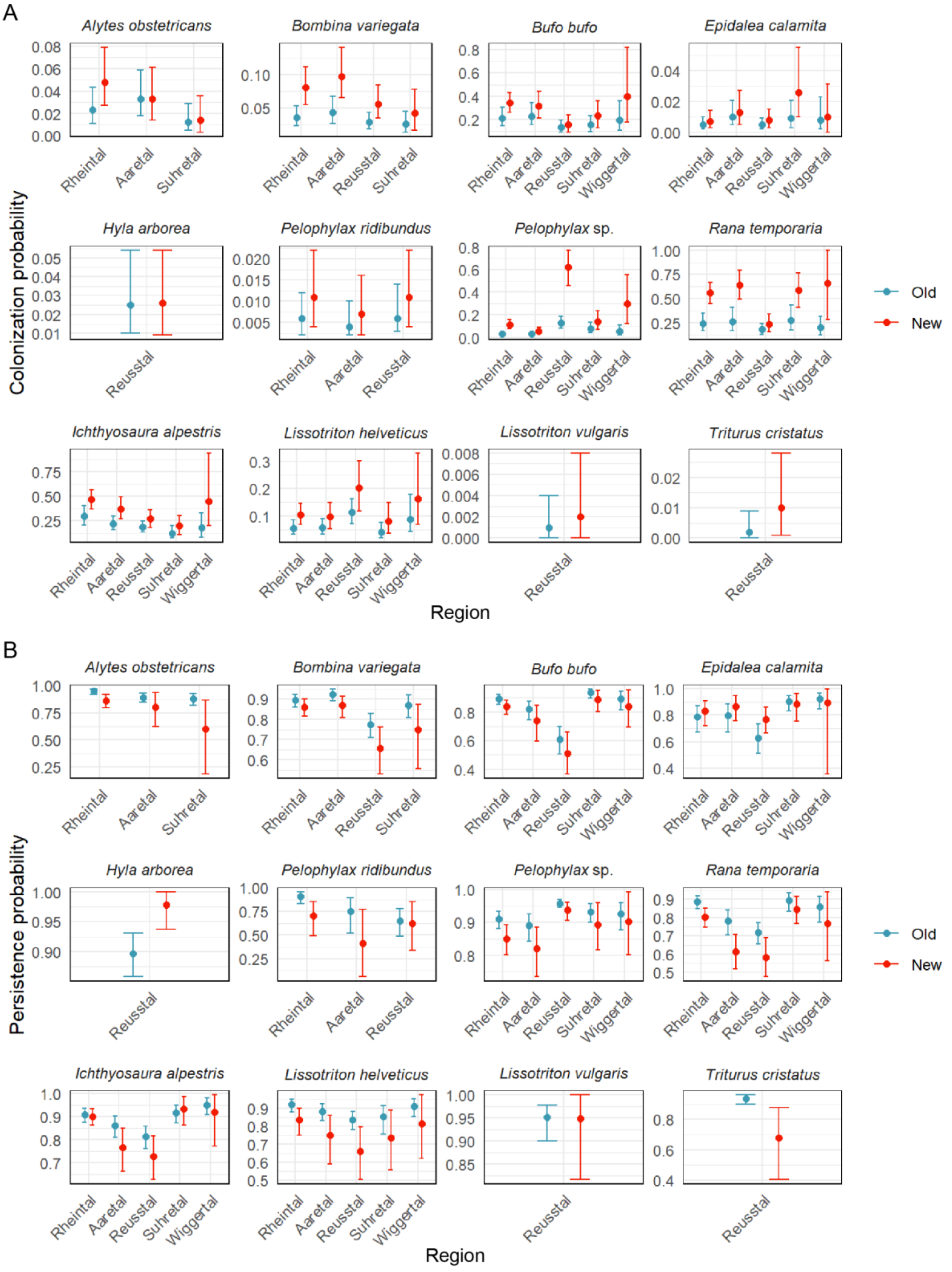

Fig. S6. Colonization (A) and persistence (B) probabilities (mean, with 95% CI) in old and new ponds in the five regions (CIs include variability in the region-specific intercept as well as variability in the region-specific effect of new ponds).

Table S4. Parameter estimates of dynamic occupancy models; bold font indicates coefficients with >95% of the posterior density above or below zero. ψ_1 is the expected mean initial occupancy probability across all regions on the probability scale, with variance σ_1^2 indicating variability across regions. All other coefficients are given on the logit scale. $\mu_{\alpha,\gamma}$ and $\mu_{\alpha,\varphi}$ are the expected mean intercepts across all regions, with variances $\sigma_{\alpha,\gamma}^2$ and $\sigma_{\alpha,\varphi}^2$. $\mu_{\beta New}$ and $\mu_{\delta New}$ are the expected mean effects of new ponds, as compared to old ponds (effects parameterization), with variances $\sigma_{\beta New}^2$ and $\sigma_{\delta New}^2$. *Hyla arborea*, *Lissotriton vulgaris*, and *Triturus cristatus* occurred only in the Reusstal, hence there is no variability in the intercept $\mu_{\alpha,\gamma}$ or the difference for new ponds $\mu_{\beta New}$.

	**Occupancy probability year1**	**Colonization**											**Persistence**	
**Species**	ψ_1 (σ_1^2)	$\mu_{\alpha,\gamma}(\sigma_{\alpha,\gamma}^2)$	$\mu_{\beta New}(\sigma_{\beta New}^2)$	**Elev**	**Area**	**Area²**	**Fluct**	**Forest**	**Forest²**	**Roads**	**Age**	**Conn**	$\mu_{\alpha,\varphi}(\sigma_{\alpha,\varphi}^2)$	$\mu_{\delta New}(\sigma_{\beta New}^2)$
A. obs.	0.34 (0.47)	-2.58 (5.7)	0.26 (0.19)	**0.72**	**0.68**	**-0.30**	0.29	0.08	**-0.47**	-0.06	0.04	-0.21	**1.81 (2.62)**	**-1.10 (0.18)**
B. var.	0.32 (0.50)	**-3.2 (0.43)**	**0.69 (0.16)**	0.02	**-0.28**	-0.01	**0.77**	**0.36**	**-0.16**	**0.17**	**-0.38**	**0.45**	**1.72 (1.31)**	**-0.58 (0.16)**
B. buf.	0.56 (0.57)	**-1.46 (0.26)**	**0.50 (0.17)**	**0.40**	**0.74**	-0.07	-0.03	**0.36**	**-0.24**	0.02	0.01	0.08	**1.54 (1.93)**	-0.44 (0.16)
E. cal.	0.18 (0.53)	**-4.73 (0.77)**	0.46 (0.18)	0.15	**0.62**	0.00	**1.00**	**-1.23**	**-0.48**	0.00	-0.04	0.04	**1.38 (1.58)**	0.37 (0.18)
H. arb.	0.1	**-3.69**	-0.02	**-1.09**	0.15	**-0.20**	0.09	-0.06	**-0.43**	**-0.87**	-0.43	**0.24**	**2.18**	**2.08**
Pel. sp.	0.42 (0.69)	**-2.52 (1.47)**	**1.33 (0.3)**	**-1.03**	**0.63**	-0.06	0.09	**0.14**	0.05	**-0.38**	0.11	-0.09	**2.42 (0.5)**	-0.43 (0.16)
P. rid.	0.17 (0.99)	**-3.97 (4.18)**	0.44 (0.17)	**-1.81**	**0.77**	-0.08	-0.11	**-0.39**	-0.16	**-0.62**	**-0.59**	-0.13	0.9 (3.83)	-0.87 (0.21)
R. tem.	0.55 (0.34)	**-1.14 (0.25)**	**1.21 (0.21)**	**0.51**	**0.39**	**-0.08**	-0.10	**0.30**	**-0.17**	**-0.16**	**-0.28**	**-0.16**	**1.51 (0.72)**	**-0.58 (0.15)**
I. alp.	0.52 (0.56)	**-1.33 (0.52)**	**0.72 (0.16)**	**0.73**	**0.13**	**-0.14**	**0.19**	**0.29**	0.04	**0.12**	-0.01	-0.05	**2.02 (1.01)**	-0.26 (0.18)
L. hel.	0.36 (0.4)	**-2.5 (0.67)**	**0.62 (0.15)**	-0.08	**0.48**	**-0.28**	**0.27**	**0.39**	**-0.19**	0.09	0.12	-0.12	**1.95 (0.4)**	**-0.79 (0.16)**
L. vul.	0.14	**-7.59**	0.75	-0.07	**0.82**	0.03	**1.65**	**-1.13**	-0.43	**-2.48**	-0.11	-0.06	**2.99**	1.61
T. cri.	0.17	**-6.03**	**1.15**	-0.62	0.36	-0.07	**0.73**	-0.39	0.03	**-2.26**	0.52	0.20	**2.71**	**-1.89**

SI Appendix S2. Costs of pond construction.

In this supplement, we briefly describe the costs of pond construction. Because ponds were built over a period of twenty years by different stakeholders, we cannot provide an exact price (35). Therefore, we provide costs for pond construction which are based on forty years of experience in Switzerland (34). Costs will be different in other places (36).

As there are several steps in pond construction, costs can be incurred at all these steps. The first step is the selection of the site. This means that repeated site visits may be necessary to discuss the pond construction project with the land owner, local authorities, farmers, foresters and others. The time used for these discussions should be included in a budget. Some technical assessments may also be necessary. For example, this may involve the determination of ground water levels. This may cost money. Depending on the site, it may also be necessary to apply for a construction permit for which authorities may charge a fee.

There are costs for pond construction. They vary a lot depending on the site, soil type, pond size, type of pond lining and other factors. In our experience, costs for pond construction can range anywhere from CHF 5’000 to 50’000 (37). The average expected cost of pond construction in the Swiss state Aargau is CHF 1’000 per 10m^2 pond surface. Pond construction costs also vary substantially in England (38).

Once the pond is constructed, it has to be managed. The costs for pond management vary depending on the pond. They may add up over the years to costs comparable to those of the initial construction.

Mermod *et al.* (39) provided two detailed examples of the steps necessary to plan and construct a pond and the associated costs. Costs are broken down into categories (e.g., labour, capital consumables; as suggested by 35). The first example of Mermod *et al.* (35) is for a pond with a size of 30x40 meters and a depth of 1 meter on wet soil without a liner. Total costs were CHF 14’491 (excluding value-added tax). The second example is for two small ponds (11x7.5 meters, depth 0.6 meters; 5x7 meters, depth 0.6 meters) in the forest; costs were CHF 11’467. These costs included the costs for pond construction itself as well as the costs for an ecological consultant who supervised pond construction. They did not cover costs for land acquisition. Similar cost calculations can be found in Pellet (37).

SI Appendix S3. Standardized field protocol of the monitoring program.

The field protocol used in the amphibian monitoring program of the Swiss state Aargau is standardized and all fieldworkers are trained in its application by a professional coordination office. The monitoring program started in 1999 with the main aim to survey trends of the eight target species of the conservation program (see **Table S1**); fieldworkers are instructed, however, to record all amphibian species present. The total extant amphibian diversity of this landscape encompasses the twelve pond-breeding species studied here (plus one salamander (*Salamandra salamandra*), which deposits its larvae in small streams).

Each year, two to three subregions (see **Fig. S1**) are surveyed comprehensively, that is every site that is potentially suitable habitat for a target species is visited. The coordination office manages the site data, selects the annual survey sites and coordinates the surveys. Each site is visited three times between April 20 and July 31. The first visit happens between April 20 and May 15, during the night; the second visit between May 16 and May 31, during the night; and the third visit between June 15 and July 31, during the day (mainly intended to detect diurnal species and tadpoles). Visits are carried out in mild and humid weather conditions.

The time spent during a visit is fixed depending on the surface area of the pond: 20 minutes observation time for total surface area 1-100 m^2; 40 minutes for 101-1'000 m^2; 60 minutes for 1'001-10'000 m^2; 90 minutes for >10'000 m^2. Observation time means the time spent actively searching for animals or listening for calls at the site. Each visit is conducted in two steps: first, the site is approached carefully, avoiding disturbance, and the number of calling individuals is recorded; second, the entire shoreline of the site is surveyed with the aid of a strong flashlight, and all visible or audible individuals are counted. The presence of larvae or eggs is also recorded (in three abundance categories). Uncertain identifications are marked as such.

The condition of each site is also recorded at each visit. Fluctuations in water level between successive visits were noted by fieldworkers in two categories: minor fluctuations and major fluctuations (the latter including complete drying of the pond). The probability of category 'major fluctuations' occurring was checked during data cleaning by comparison with the water surface area (smaller ponds were more likely to have strongly fluctuating water levels) and the type of the pond (e.g., shallow ponds in gravel pits were more likely to have strongly fluctuating water tables than larger, deeper pools in the backwater of a river). In case of doubt, we assumed 'minor fluctuations'.

To minimize variability between observers, we conservatively aggregated counts and abundance categories to presence-absence data per site. Observer variability in detection probability was further accounted for by means of a random effect for observer identity in the observation model. Uncertain identifications were excluded. False positive errors (misidentifications) were not modelled, but have been shown to be negligible in our study system, especially for rare species. False positive errors had no effect on temporal trends (40).

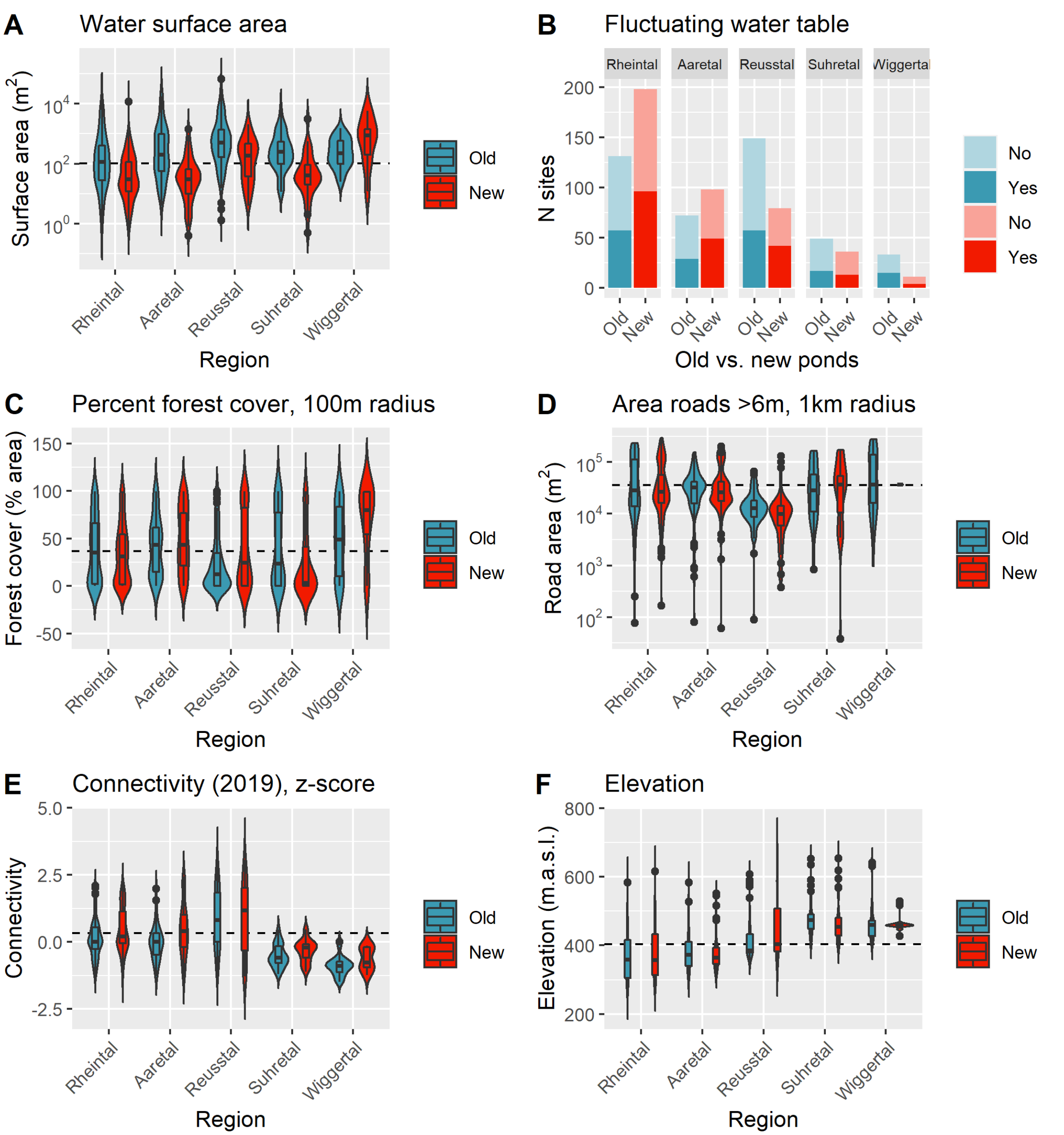

Fig. S7. Distribution of covariate values in old and new ponds, in the five regions, with grand mean indicated by the dashed line. (A) The water surface area of ponds was below average in Rheintal (β = -0.3, 95% CI [-0.4, -0.2], p<0.001) and Aaretal (β = -0.16, 95% CI [-0.3, -0.03], p=0.02), and above average in Reusstal (β = 0.5, 95% CI [0.3, 0.6], p<0.001) and Wiggertal (β = 0.5, 95% CI [0.2, 0.7], p<0.01). Water surface area was smaller in new than in old ponds in all regions except in Wiggertal (Welch's t test; Rheintal: t(241)= 5.36, p<0.001; Aaretal: t(136)=6.88 , p<0.001; Reusstal t(164)= 5.34, p<0.001; Suhretal t(69)= 5.02, p<0.001). (B) The proportion of ponds with fluctuating water tables was below average in Reusstal (β = -0.3, 95% CI [-0.5, -0.01], p=0.048) and in Suhretal (β = -0.6, 95% CI [-1, -0.2], p<0.01). The proportion of ponds with fluctuating water tables did not differ between old and new ponds except in Reusstal, where new ponds had a higher proportion of fluctuating water tables (Binomial glm; odds ratio = 1.83, 95% CI [0.06, 2.18]). (C) The proportion of forest around ponds was below average in Rheintal (β = -0.6, 95% CI [-0.8, -0.32], p<0.001), Reusstal (β = -0.9, 95% CI [-1.2, -0.6], p<0.001), and Suhretal (β = -0.7, 95% CI [-1.2, -0.3], p<0.01). The proportion of forest around ponds did not differ between old

and new ponds except in Reusstal, where new ponds had roughly twice as much forest in their surroundings (Binomial glm; odds ratio = 2.07, 95% CI [1.15, 3.74]). (D) The area of large roads around ponds was below average in Reusstal ($\beta = -0.5$, 95% CI [-0.6, -0.3], $p<0.001$), and above average in Aaretal ($\beta = 0.3$, 95% CI [0.1, 0.4], $p<0.001$), Suhretal ($\beta = 0.2$, 95% CI [0.01, 0.4], $p=0.048$), and Wiggertal ($\beta = 0.4$, 95% CI [0.1, 0.7], $p<0.01$). The area of large roads around ponds only differed between old and new ponds in Wiggertal, where new ponds were built where there were fewer roads (Welch's t test; Wiggertal: $t(41)= 3.77$, $p<0.001$). (E) Connectivity was above average in Rheintal ($\beta = 0.3$, 95% CI [0.2, 0.4], $p<0.001$), Aaretal ($\beta = 0.2$, 95% CI [0.1, 0.4], $p<0.001$), and Reusstal ($\beta = 0.9$, 95% CI [0.7, 1], $p<0.001$). In contrast, connectivity was below average in Suhretal ($\beta = -0.4$, 95% CI [-0.6, -0.2], $p<0.001$) and Wiggertal ($\beta = -0.8$, 95% CI [-1, -0.6], $p<0.001$). Connectivity was higher for newly built ponds in Rheintal (Welch's t test, $t(302)= -3.56$, $p<0.001$), Aaretal ($t(167)= -3.76$, $p<0.001$), and Suhretal ($t(77)= -2.08$, $p=0.04$), but not in Reusstal and Wiggertal. (F) Ponds in Rheintal ($\beta = -0.4$, 95% CI [-0.5, -0.3], $p<0.001$) and Aaretal ($\beta = -0.3$, 95% CI [[-0.5, -0.2], $p<0.001$) were at lower than average elevation, while those in Suhretal ($\beta = 0.9$, 95% CI [0.8, 1.1], $p<0.001$) and Wiggertal ($\beta = 0.8$, 95% CI [0.5, 1], $p<0.001$) were at higher than average elevation. Elevation did not differ between old and new ponds except in Reusstal, where new ponds were built at higher elevations than old ponds (Welch's t test, $t(103)= -3.81$, $p<0.001$). Correlations between covariates did not exceed $r=0.35$ (a weak positive correlation between the proportion of forest around ponds and elevation).

Table S5. Parameter estimates of observation model, on the logit scale. Bold font indicates coefficients with >95% of posterior density above or below zero. The estimated variance between observers σ^2_{Obs} was high and therefore important to account for. The right-hand columns show the corresponding average detection probability at night and during the day (with 95% CI).

Species	α_p	β_{Day}	σ^2_{Obs}	Mean *p* at night	Mean *p* during day
A. obstetricans	**1.02**	**-1.13**	**1.31**	0.73 [0.65, 0.8]	0.47 [0.29, 0.66]
B. variegata	**0.83**	**-0.44**	**0.64**	0.70 [0.65, 0.74]	0.60 [0.49, 0.7]
B. bufo	**0.27**	**-1.64**	**2.14**	0.57 [0.49, 0.63]	0.20 [0.12, 0.31]
E. calamita	**0.85**	**-1.16**	**0.3**	0.70 [0.64, 0.75]	0.42 [0.3, 0.55]
H. arborea	**1.64**	**-3.34**	**1.93**	0.84 [0.75, 0.9]	0.16 [0.05, 0.36]
Pelophylax sp.	**1.61**	**-0.33**	**0.76**	0.83 [0.8, 0.86]	0.78 [0.7, 0.85]
P. ridibundus	0.003	-0.37	**3.76**	0.50 [0.3, 0.69]	0.41 [0.12, 0.78]
R. temporaria	**0.91**	**-1.63**	**0.84**	0.66 [0.62, 0.71]	0.28 [0.21, 0.37]
I. alpestris	**0.85**	**-1.18**	**0.99**	0.70 [0.66, 0.74]	0.42 [0.32, 0.52]
L. helveticus	-0.25	**-1.05**	**2.01**	0.44 [0.36, 0.52]	0.21 [0.12, 0.35]
L. vulgaris	**-0.99**	-0.03	**1.03**	0.27 [0.17, 0.37]	0.26 [0.11, 0.5]
T. cristatus	**-0.64**	-0.54	**2.82**	0.35 [0.2, 0.49]	0.24 [0.07, 0.54]

SI Appendix S4. Goodness-of-fit: Posterior predictive checks.

We assessed goodness-of-fit of the models with posterior predictive checks (41, 42). The procedure predicts new data Y_{rep} that we would expect to see under the model for each draw of the joint posterior distribution of all model parameters. Since our data Y are binary, standard fit statistics are proportional to sample size and therefore uninformative of model fit. Instead predictions need to be aggregated for comparisons between predicted and observed Y (43). We chose to aggregate Y_{rep} to the number of ponds per core area with at least one detection during years in which core areas were surveyed comprehensively. Each of the ten core areas was surveyed on average 5.5 times. Species occurring only in Reusstal hence had the fewest aggregated observations (n=15 comprehensive surveys of 3 core areas), while species occurring in all five regions (i.e. all ten core areas) had the most aggregated observations (n=55) available for comparison.

The models fit the data well (**Fig. S6**). In most cases, the predicted number of sites with at least one detection was within a range of ±2 of the actual number observed to be occupied. The largest deviations (+5 to -3 sites above and below the observed, respectively) occurred for *Lissotriton vulgaris* (a species with few observations and hence larger uncertainties) and *Rana temporaria* in one region (Aaretal).

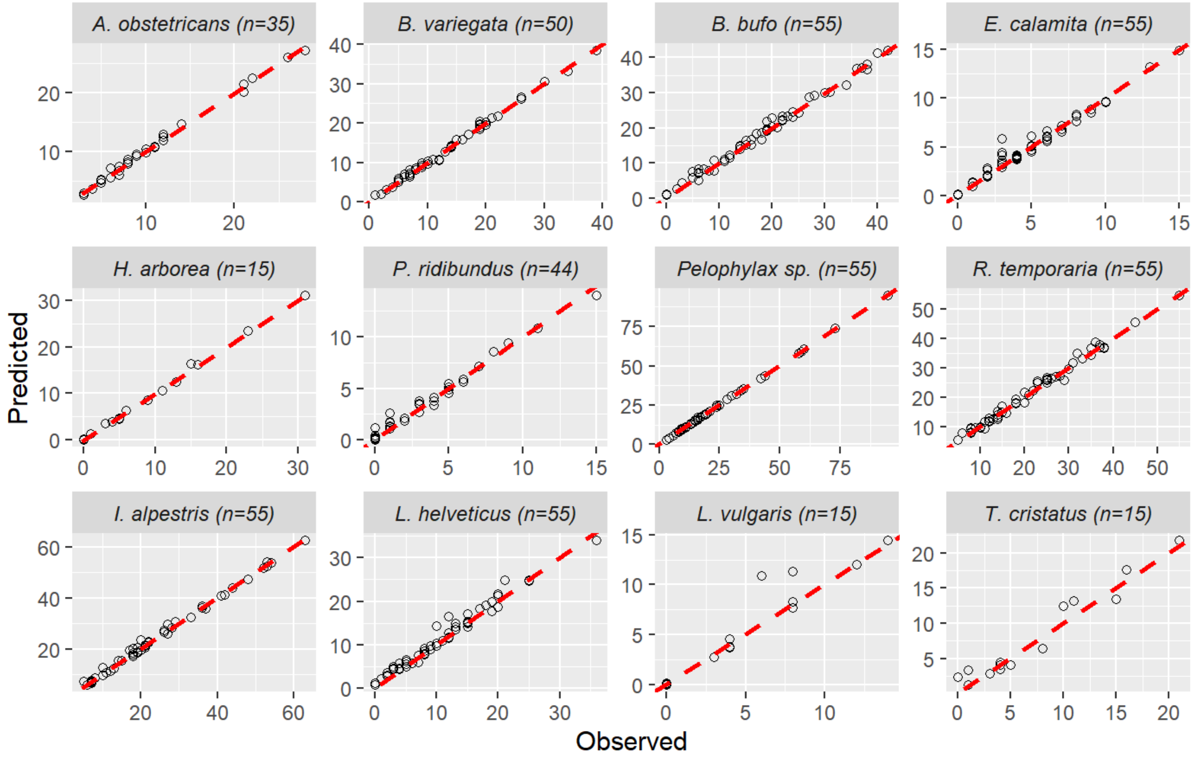

Fig. S8. Predicted number of sites with at least one detection (mean of posterior distributions of size n=1200) vs. observed number of sites with at least one detection (during comprehensive core area surveys). The number n next to species names gives the number of comprehensive subregion surveys available for comparison.

SI Appendix S5. JAGS model code.

```
Indata:
nregions     Number of regions modelled
nsite        Number of sites
nyear        Number of years
nobservers   Number of unique observers
region[i]    Site i: index of region
oldnew[i]    Site i factor: old (1) or new (2) pond
Water[i]     Site i: water surface area
Fluct[i]     Site i: water table fluctuating (1) or not fluctuating (0)
Elev[i]      Site i: elevation
Forest[i]    Site i: Proportion of forest in 100m radius
Roads[i]     Site i: Area of roads in 1km radius
extant[i,k]          Site i in year k existing? (0,1)
pond.age[i,k]        Age of site i in year k
Xconn[i,k]           Connectivity of site i in year k
detectable[i,j,k]    Was site i visited during visit j in year k? (0,1)
observer[i,j,k]      Identity of observer in site i during visit j in year k
y[i,j,k]             Detection-nondetection data for site i during visit j in
year k
```

A) JAGS model for species occurring in more than one region:

```
  model {
    ###### Specify priors ##########################################
    ### Occupancy probability in year 1:
    ## psi1: common intercept with region-specific error, on logit scale:
    alpha1.prob ~ dunif(0,1) # prior on probability scale
    alpha1 <- log(alpha1.prob / (1- alpha1.prob)) # alpha1 on logit scale
    for(a in 1:nregions){
       psi.error[a] ~ dnorm(0, precision)
    }
    # hyperparameters:
    precision <- pow(sigma, -2) # 1/sigma^2
    sigma ~ dunif(0,10) #

    ### region-specific parameters:
    for(a in 1:nregions){
       # region-specific intercepts:
      alpha.gamma[a]~ dnorm(mu.alpha.gamma, tau.alpha.gamma) # logit scale
      alpha.phi[a] ~ dnorm(mu.alpha.phi, tau.alpha.phi) # logit scale
      # region-specific effect of new ponds ponds (effects parameterization):
      beta.oldnew[a,1] <- 0 # old ponds are baseline
      beta.oldnew[a,2] ~ dnorm(mu.gamma.new, tau.gamma.new)
      delta.oldnew[a,1] <- 0 # old ponds are baseline
      delta.oldnew[a,2] ~ dnorm(mu.phi.new, tau.phi.new)
    }

    # hyperparameters for random intercepts:
    mu.alpha.gamma <- log(mu.alpha.gamma.prob / (1 -mu.alpha.gamma.prob  ))
    mu.alpha.gamma.prob ~ dunif(0,1) # prior on probability scale
    tau.alpha.gamma <- 1 / (sigma.alpha.gamma * sigma.alpha.gamma)
    sigma.alpha.gamma ~ dunif(0,10)

    mu.alpha.phi <- log(mu.alpha.phi.prob / (1 -mu.alpha.phi.prob  ))
    mu.alpha.phi.prob ~ dunif(0,1)
    tau.alpha.phi <- 1 / (sigma.alpha.phi * sigma.alpha.phi)
    sigma.alpha.phi ~ dunif(0,10)

    # hyperparameters for effect of new ponds:
```

```
mu.gamma.new ~ dunif(-10,10)
tau.gamma.new <- 1 / (sigma.gamma.new * sigma.gamma.new)
sigma.gamma.new ~ dunif(0,10)

mu.phi.new ~ dunif(-10,10)
tau.phi.new <- 1 / (sigma.phi.new * sigma.phi.new)
sigma.phi.new ~ dunif(0,10)

### Priors for betas (fixed effects coefficients, logit scale):
beta.water~dunif(-10,10)
beta.water.2~dunif(-10,10)
beta.fluct~dunif(-10,10)
beta.elev~dunif(-10,10
beta.forest~dunif(-10,10)
beta.forest.2~dunif(-10,10)
beta.roads~dunif(-10,10)
beta.age~dunif(-10,10)
beta.conn~dunif(-10,10)

### Pdet parameters:
det.int.prob ~ dunif(0,1) # on probability scale, detection mean
det.int <- log(det.int.prob / (1 - det.int.prob)) # logit
day.const ~ dnorm(0,0.01)  # Day constant

# Random observer effects:
for (obs in 1:nobservers){
  beta.obs[obs] ~ dnorm(0, tau.beta.obs)
}
tau.beta.obs <- 1/ (sd.beta.obs * sd.beta.obs)
sd.beta.obs ~ dunif(0,10)

##### Ecological submodel: #########################################
for (i in 1:nsite){
    # Initial occupancy:
    logit(psi1[i]) <- alpha1 + psi.error[region[i]]
    z[i,1] ~ dbern(psi1[i]*extant[i,1])

    # Persistence phi: (constant through time)
   logit(phi[i]) <- alpha.phi[region[i]] +
delta.oldnew[region[i],oldnew[i]]

  for (k in 1:(nyear-1)){
     # Colonization gamma:
     logit(gamma[i,k]) <- alpha.gamma[region[i]] +
   beta.oldnew[region[i],oldnew[i]] + beta.water*Water[i] + beta.water.2*
   pow(Water[i],2) + beta.fluct*Fluct[i]+ beta.elev*Elev[i]+
   beta.forest*Forest[i] + beta.forest.2 * pow(Forest[i],2) +
   beta.roads*Roads[i] + beta.age * pond.age[i,k] + beta.conn * Xconn[i,k]
     # State in year t+1 as function of colonization and persistence:
     muZ[i,(k+1)]<- (z[i,k]*phi[i]) + ((1-z[i,k])*gamma[i,k])
     z[i,(k+1)] ~ dbern(muZ[i,(k+1)]*extant[i,(k+1)])
    } #k
} #i

##### Observation model:#########################################
# Detection probability p:
# Separate loop over sites and years for p components
for (i in 1:nsite){
      for (k in 1:nyear){
        logit(p[i,1,k]) <- det.int + beta.obs[observer[i,1,k]] # night
        logit(p[i,2,k]) <- det.int + beta.obs[observer[i,2,k]] # night
        logit(p[i,3,k]) <- det.int + day.const # third visit during day
```

```
        }
      }
    for (i in 1:nsite){
        for (j in 1:nrep){
            for (k in 1:nyear){
           # relate observations y to true state z:
            muy[i,j,k] <- z[i,k]*p[i,j,k] * detectable[i,j,k]
            y[i,j,k] ~ dbern(muy[i,j,k])
          } #k
        } #j
    } #i

} ## end model
################################################################################
####
```

B) JAGS model for species occurring in only one region:

```
    model {
    ###### Specify priors ##########################################
    ### Occupancy probability in year 1:
    ## psi1: (single area
    alpha1.prob ~ dunif(0,1) # prior on probability scale
    alpha1 <- log(alpha1.prob / (1- alpha1.prob)) # alpha1 on logit scale

    ### Intercepts for single region:
    alpha.gamma ~ dunif(-10,10) # on logit scale
    alpha.phi ~ dunif(-10,10) # on logit scale

    ### Effect of new ponds (effects parameterization):
    beta.oldnew[1] <- 0
    beta.oldnew[2] ~ dunif(-10,10)

    delta.oldnew[1] <- 0
    delta.oldnew[2] ~ dunif(-10,10)

    ### Priors for betas (fixed effects coefficients, logit scale):
    beta.water~dunif(-10,10)
    beta.water.2~dunif(-10,10)
    beta.fluct~dunif(-10,10)
    beta.elev~dunif(-10,10)
    beta.forest~dunif(-10,10)
    beta.forest.2~dunif(-10,10)
    beta.roads~dunif(-10,10)
    beta.age~dunif(-10,10)
    beta.conn~dunif(-10,10)

    ### Pdet parameters:
    det.int.prob ~ dunif(0,1) # on probability scale, detection mean
    det.int <- log(det.int.prob / (1 - det.int.prob)) # logit
    day.const ~ dnorm(0,0.01)  # Day constant

    # Random observer effects:
    for (obs in 1:nobservers){
      beta.obs[obs] ~ dnorm(0, tau.beta.obs)
    }
    tau.beta.obs <- 1/ (sd.beta.obs * sd.beta.obs)
    sd.beta.obs ~ dunif(0,10)

    ##### Ecological submodel: ########################################
    for (i in 1:nsite){
```

```
        # Initial occupancy:
        logit(psi1[i]) <- alpha1
        z[i,1] ~ dbern(psi1[i]*extant[i,1])

        # Persistence phi: (constant through time)
        logit(phi[i]) <- alpha.phi + delta.oldnew[oldnew[i]]

     for (k in 1:(nyear-1)){
         # Colonization gamma:
         logit(gamma[i,k]) <- alpha.gamma + beta.oldnew[oldnew[i]] +
beta.water*Water[i] + beta.water.2* pow(Water[i],2) + beta.fluct*Fluct[i]+
beta.elev*Elev[i]+ beta.forest*Forest[i] + beta.forest.2 * pow(Forest[i],2) +
beta.roads*Roads[i] + beta.age * pond.age[i,k] + beta.conn* Xconn[i,k]
         # State in year t+1 as function of colonization and persistence:
         muZ[i,(k+1)]<- (z[i,k]*phi[i]) + ((1-z[i,k])*gamma[i,k])
         z[i,(k+1)] ~ dbern(muZ[i,(k+1)]*extant[i,(k+1)])
        } #k
    } #i

    ##### Observation model:########################################
    # Detection probability p:
    # Separate loop over sites and years for p components
    for (i in 1:nsite){
          for (k in 1:nyear){
            logit(p[i,1,k]) <- det.int + beta.obs[observer[i,1,k]]
            logit(p[i,2,k]) <- det.int + beta.obs[observer[i,2,k]]
            logit(p[i,3,k]) <- det.int + day.const
           }
      }
    for (i in 1:nsite){
        for (j in 1:nrep){
            for (k in 1:nyear){
            muy[i,j,k] <- z[i,k]*p[i,j,k] *detectable[i,j,k]
            y[i,j,k] ~ dbern(muy[i,j,k])
            # predict y.rep:
            y.new[i,j,k] ~ dbern(muy[i,j,k])
            } #k
        } #j
    } #i

} # end model
################################################################################
```

SI References

1. A. Trochet, *et al.*, A database of life-history traits of European amphibians. *Biodivers. Data J.* **2** (2014).
2. S. S. Cruickshank, A. Ozgul, S. Zumbach, B. R. Schmidt, Quantifying population declines based on presence-only records for red-list assessments. *Conserv. Biol.* **30**, 1112–1121 (2016).
3. A. Meyer, S. Zumbach, B. R. Schmidt, J.-C. Monney, *Auf Schlangenspuren und Krötenpfaden. Amphibien und Reptilien der Schweiz.* (Haupt Verlag, 2009).
4. C. C. Geiger, C. Bregnard, E. Maluenda, M. J. Voordouw, B. R. Schmidt, Antifungal treatment of wild amphibian populations caused a transient reduction in the prevalence of the fungal pathogen, *Batrachochytrium dendrobatidis*. *Sci. Rep.* **7**, 1–12 (2017).
5. J. Van Buskirk, Habitat partitioning in European and North American pond-breeding frogs and toads. *Divers. Distrib.* **9**, 399–410 (2003).
6. T. Roth, C. Bühler, V. Amrhein, Estimating effects of species interactions on populations of endangered species. *Am. Nat.* **187**, 457–467 (2016).
7. D. C. Woodhams, *et al.*, Interacting symbionts and immunity in the amphibian skin mucosome predict disease risk and probiotic effectiveness. *PLoS One* **9** (2014).
8. S. S. Cruickshank, A. Bergamini, B. R. Schmidt, Estimation of breeding probability can make monitoring data more revealing: a case study of amphibians. *Ecol. Appl.* **31**, 1–12 (2021).
9. R. Jehle, U. Sinsch, Wanderleistung und Orientierung von Amphibien: eine Übersicht. *Z. Feldherpetol.* **14**, 137–152 (2007).
10. A. Morand, P. Joly, Habitat variability and space utilization by the amphibian communities of the French Upper-Rhone floodplain. *Hydrobiologia* **300/301**, 249–257 (1995).
11. H. Barandun, H. Reyer, Reproductive ecology of *Bombina variegata*: aspects of life history. *Amphib. Reptil.* **18**, 347–355 (1997).
12. H. Cayuela, S. S. Cruickshank, H. Brandt, A. Ozgul, B. R. Schmidt, Habitat-driven life history variation in an amphibian metapopulation. *Oikos* **128**, 1265–1276 (2019).
13. H. Cayuela, *et al.*, Anthropogenic disturbance drives dispersal syndromes, demography, and gene flow in amphibian populations. *Ecol. Monogr.* **90**, 1–25 (2020).
14. S. O. Petrovan, B. R. Schmidt, Volunteer conservation action data reveals large-scale and long-term negative population trends of a widespread amphibian, the common toad (*Bufo bufo*). *PLoS One* **11**, e0161943 (2016).
15. J. Van Buskirk, Local and landscape influence on amphibian occurrence and abundance. *Ecology* **86**, 1936–1947 (2005).
16. J. Van Buskirk, Permeability of the landscape matrix between amphibian breeding sites. *Ecol. Evol.* **2**, 3160–3167 (2012).
17. L. Indermaur, M. Schaub, J. Jokela, K. Tockner, B. R. Schmidt, Differential response to abiotic conditions and predation risk rather than competition avoidance determine breeding site selection by anurans. *Ecography (Cop.).* **33**, 887–895 (2010).
18. F. Zanini, J. Pellet, B. R. Schmidt, The transferability of distribution models across regions: An amphibian case study. *Divers. Distrib.* **15**, 469–480 (2009).
19. B. R. Schmidt, J. Pellet, Relative importance of population processes and habitat characteristics in determining site occupancy of two anurans. *J. Wildl. Manage.* **69**, 884–893 (2005).
20. U. Sinsch, N. Oromi, C. Miaud, J. Denton, D. Sanuy, Connectivity of local amphibian populations: Modelling the migratory capacity of radio-tracked natterjack toads. *Anim. Conserv.* **15**, 388–396 (2012).
21. A. Carlson, P. Edenhamn, Extinction dynamics and the regional persistence of a tree frog metapopulation. *Proc. R. Soc. B Biol. Sci.* **267**, 1311–1313 (2000).
22. T. Broquet, J. Jaquiéry, N. Perrin, Opportunity for sexual selection and effective population size in the lek-breeding European treefrog (*Hyla arborea*). *Evolution (N. Y).* **63**, 674–683 (2009).

23. C. Vorburger, Non-hybrid offspring from matings between hemiclonal hybrid waterfrogs suggest occasional recombination between clonal genomes. *Ecol. Lett.* **4**, 628–636 (2001).
24. S. Dubey, J. Leuenberger, N. Perrin, Multiple origins of invasive and “native” water frogs (*Pelophylax* spp.) in Switzerland. *Biol. J. Linn. Soc.* **112**, 442–449 (2014).
25. C. Dufresnes, *et al.*, Invasion genetics of marsh frogs (*Pelophylax ridibundus* sensu lato) in Switzerland. *Biol. J. Linn. Soc.* **123**, 402–410 (2018).
26. B. R. Anholt, S. Negovetic, C. Rauter, C. Som, Predator complement determines the relative success of tadpoles of the *Rana esculenta* complex. *Evol. Ecol. Res.* **7**, 733–741 (2005).
27. K. Grossenbacher, *Verbreitungsatlas der Amphibien der Schweiz* (Schweizerischer Bund für Naturschutz, 1988).
28. B. R. Schmidt, S. Zumbach, *Rote Liste der gefährdeten Arten der Schweiz: Amphibien* (Bundesamt für Umwelt, Wald und Landschaft, 2005).
29. H. Luqman, *et al.*, No distinct barrier effects of highways and a wide river on the genetic structure of the Alpine newt (*Ichthyosaura alpestris*) in densely settled landscapes. *Conserv. Genet.* **19**, 673–685 (2018).
30. M. Denoël, A. Lehmann, Multi-scale effect of landscape processes and habitat quality on newt abundance: Implications for conservation. *Biol. Conserv.* **130**, 495–504 (2006).
31. B. Unglaub, S. Steinfartz, A. Drechsler, B. R. Schmidt, Linking habitat suitability to demography in a pond-breeding amphibian. *Front. Zool.* **12**, 1–10 (2015).
32. B. Unglaub, *et al.*, Context-dependent dispersal determines relatedness and genetic structure in a patchy amphibian population. *Mol. Ecol.* **30**, 5009–5028 (2021).
33. B. R. Schmidt, J. Van Buskirk, A comparative analysis of predator-induced plasticity in larval *Triturus* newts. *J. Evol. Biol.* **18**, 415–425 (2005).
34. B. R. Schmidt, S. Zumbach, “Amphibian conservation in Switzerland” in *Amphibian Biology, Volume 11: Status of Conservation and Decline of Amphibians: Eastern Hemisphere, Part 5: Northern Europe.*, H. Heatwole, J. W. Wilkinson, Eds. (Pelagic Publishing, 2019), pp. 46–51.
35. T.B. White, S.O. Petrovan, A.P. Christie, P.A. Martin, W.J. Sutherland. What is the price of conservation? A review of the status quo and recommendations for improving cost reporting. *BioScience* **72**, 461–471 (2022).
36. A. Balmford, K.J. Gaston, S. Blyth, A. James, Kapos V., Global variation in terrestrial conservation costs, conservation benefits, and unmet conservation needs. *Proc. Natl. Acad. Sci. U.S.A.* **100**, 1046–1050 (2003).
37. J. Pellet, Temporäre Gewässer für gefährdete Amphibien schaffen - Leitfaden für die Praxis. *Beiträge zum Naturschutz in der Schweiz* **35**, 1–25 (2014).
38. Freshwater Habitats Trust, *Pond creation toolkit sheet 7* (Freshwater Habitats Trust, Oxford, 2011). Download at https://freshwaterhabitats.org.uk/wp-content/uploads/2013/09/MPP-Toolkit-core-sheets-1-8_June2011.pdf (accessed 8th August 2022).
39. M. Mermod, S. Zumbach, A. Borgula, E. Krummenacher, B. Lüscher, J. Pellet, B.R. Schmidt*, Praxismerkblatt Artenschutz Kreuzkröte Bufo calamita* (info fauna karch, Neuchâtel, Switzerland, 2010). Download at http://karch.ch/karch/de/home/amphibien-fordern/praxismerkblatter.html (accessed 8th August 2022).
40. S. S. Cruickshank, C. Bühler, B. R. Schmidt, Quantifying data quality in a citizen science monitoring program: False negatives, false positives and occupancy trends. *Conserv. Sci. Pract.* **1**, e54 (2019).
41. A. Gelman, J. B. Carlin, H. S. Stern, D. B. Rubin, *Bayesian Data Analysis*, 2nd Ed. (Chapman & Hall/CRC, 2004).
42. D. I. MacKenzie, *et al.*, *Occupancy estimation and modeling. Inferring patterns and dynamics of species occurrence*, 2nd Edition (Elsevier Academic Press Inc, 2018).
43. M. Kéry, J. A. Royle, *Applied Hierarchical Modeling in Ecology. Analysis of distribution, abundance and species richness in R and BUGS. Volume 1* (Elsevier Academic Press Inc, 2016).
